# First validation of a novel assessgame quantifying selective voluntary motor control in children with upper motor neuron lesions

**DOI:** 10.1038/s41598-019-56495-8

**Published:** 2019-12-30

**Authors:** Jeffrey W. Keller, Julia Balzer, Annina Fahr, Jan Lieber, Urs Keller, Hubertus J. A. van Hedel

**Affiliations:** 1 Rehabilitation Center for Children and Adolescents, University Children’s Hospital Zurich, Affoltern am Albis, Switzerland; 2 Children’s Research Center, University Children’s Hospital Zurich, Zurich, Switzerland; 3 Doctoral Program Clinical Science, Faculty of Medicine, University of Zurich, Zurich, Switzerland; 4 Centre for Health, Activity and Rehabilitation Research, Queen Margaret University, Edinburgh, Scotland

**Keywords:** Diagnosis, Paediatric research

## Abstract

The question whether novel rehabilitation interventions can exploit restorative rather than compensatory mechanisms has gained momentum in recent years. Assessments measuring selective voluntary motor control could answer this question. However, while current clinical assessments are ordinal-scaled, which could affect their sensitivity, lab-based assessments are costly and time-consuming. We propose a novel, interval-scaled, computer-based assessment game using low-cost accelerometers to evaluate selective voluntary motor control. Participants steer an avatar owl on a star-studded path by moving the targeted joint of the upper or lower extremities. We calculate a target joint accuracy metric, and an outcome score for the frequency and amplitude of involuntary movements of adjacent and contralateral joints as well as the trunk. We detail the methods and, as a first proof of concept, relate the results of select children with upper motor neuron lesions (n = 48) to reference groups of neurologically intact children (n = 62) and adults (n = 64). Linear mixed models indicated that the cumulative therapist score, rating the degree of selectivity, was a good predictor of the involuntary movements outcome score. This highlights the validity of this assessgame approach to quantify selective voluntary motor control and warrants a more thorough exploration to quantify changes induced by restorative interventions.

## Introduction

After an upper motor neuron lesion, patients often exhibit multiple symptoms contributing to their disability. One possibility to classify upper motor neuron symptoms is to differentiate between positive and negative motor signs. Positive motor signs describe increased magnitude and frequency of involuntarily muscle activations, such as hypertonia and tremor. Negative motor signs are characterized by insufficient muscle activity or impaired control thereof. Examples are weakness, reduced selective voluntary motor control, ataxia, and apraxia^1^.

Unilateral or bilateral spastic cerebral palsy, but also acquired brain lesions like after stroke or traumatic brain injury, can be considered typical examples of upper motor neuron disorders where patients exhibit these positive and negative motor signs^1,2^. Even though negative motor signs may contribute more to a child’s disability than the positive ones^2,3^, it is more difficult to quantify them^1^. Of the various motor signs, reduced selective voluntary motor control (SVMC) was found to be one of the main determinants of a less favorable course of gross motor function in children with cerebral palsy^4,5^. SVMC has been defined as *“the ability to isolate the activation of muscles in a selected pattern in response to demands of a voluntary posture or movement”*^1^. Thereby, SVMC includes the isolated activation of large muscles but also small ones required for accurate fine-motor dexterous movements in absence of unusual movement patterns and postures. A reduction in SVMC manifests itself in different ways, such as reduced motor control and movements of the contralateral side^6^, known as mirror movements, muscle synergy patterns^7^ affecting multiple joints, and other involuntary movements that can involve, for example, the trunk^8^. This highlights that objectively capturing motor control and the multitude involuntary movements is quite demanding for an assessment.

However, such an assessment could answer the question whether certain interventions can exploit restorative rather than compensatory mechanisms in patients with upper motor neuron lesions, which has gained momentum in recent years^9–11^. Various novel interventions claim to do exactly this. In cerebral palsy, for example, clinical trials are underway to investigate the efficacy and safety of regenerative or neuroprotective interventions such as stem cells derived from umbilical cord blood cells^12^. Furthermore, robotic systems have been developed that aim to induce physiological, selective movements and simultaneously provide an environment where many repetitions can be performed in a playful and motivating way by using exergames^13^. These systems^14–17^ and other assistive devices^18^, as well as a motivational gestural feedback robot^19^ were evaluated to assess, in some form, their ability to promote restorative mechanisms. The same has been done for commercially available systems like the PlayStation^20^, Xbox^21^, and Nintendo Wii^22^. Moreover, systems using surface electromyography (sEMG) have been used to train selective muscle activation^23,24^. In their study, Rios *et al*.^24^ additionally used sEMG to detect unwanted co-activation of other muscles during isolated as well as functional movements. Last but not least, conventional physiotherapy concepts such as neurodevelopmental therapy aim to improve, amongst others, selective motor control^25,26^.

To detect therapy-induced changes in SVMC or such occurring spontaneously after acquired upper motor neuron lesions, practicable, valid, reliable, and responsive outcome measures are needed. Clinical assessments aiming to evaluate SVMC seek to identify adequate movement responses of the target muscle groups, while involuntary movements in other joints, body segments (e.g. the trunk), mirror and mass movement patterns need to be quantified^1,27–29^. However, in cerebral palsy for example, few assessments were designed specifically to measure SVMC of the lower^30^ and upper limbs^31,32^, and this is also the case for other neuromotor disorders. The clinical assessment tools that evaluate SVMC are practical to apply but quantify SVMC on a somewhat subjective, ordinal scale. In cerebral palsy, examples include the “Selective Control of Upper the Extremity Scale” (SCUES)^28^, or the “Selective Control Assessment of the Lower Extremity” (SCALE)^27^. More laboratory-based approaches such as surface electromyography^33^ (for an overview see^30^) require costly equipment, trained assessors, and considerable time for assessment and analysis.

The goal of this methodological paper is to present a playful assessment (analogous to the term ‘exergame’, we introduce the term ‘assessgame’) based on accelerometer measurements. The game was designed to measure SVMC of both lower and upper extremities objectively on an interval scale. We present the setup of the assessgame and the algorithms used to process and analyze the data. Furthermore, we provide first proof of concept by illustrating the interpretation of the data by comparing data of individual pediatric patients with upper motor neuron lesions to data of neurologically intact peers and adults. Finally, we examine how measures of SVMC, severity of impairment, and age relate to our assessgame outcome. Later studies will evaluate the applicability of this approach in other patient groups with upper motor neuron lesions and determine the psychometric properties of this assessgame for objectively quantifying SVMC of the lower and upper limbs.

## Results

### Properties of the assessgame

**Figure 1:**
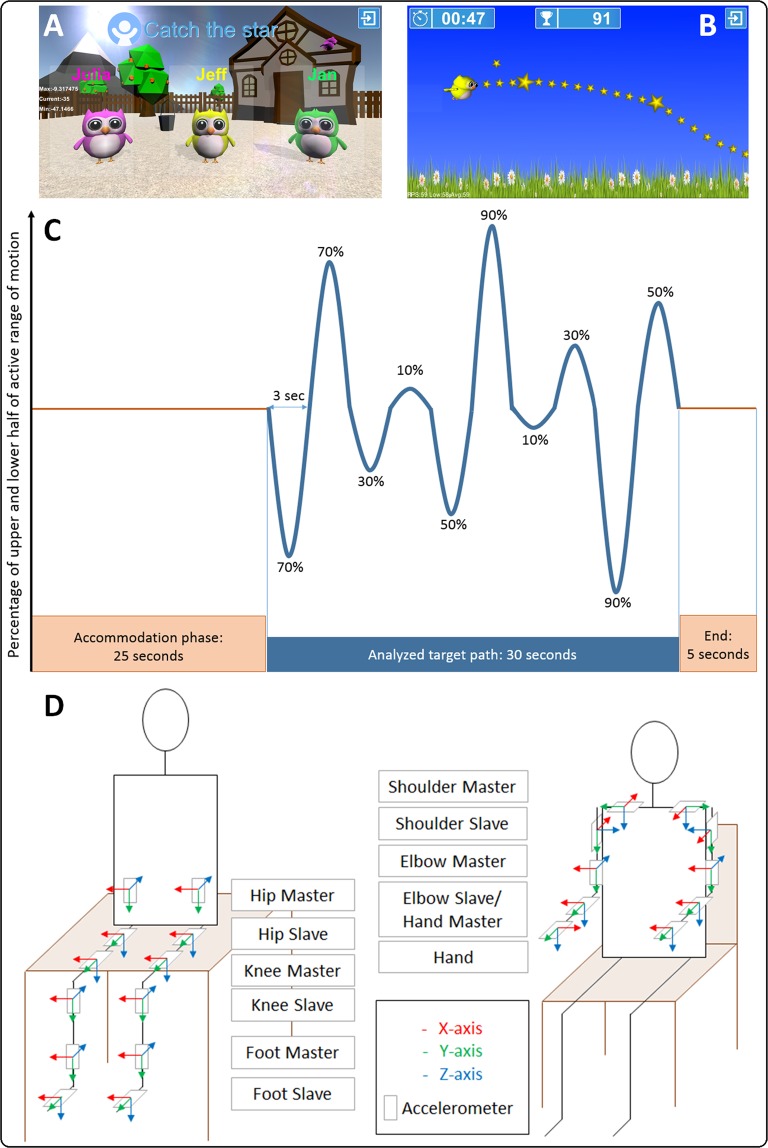
Assessgame elements and sensor placement. (**A**) Start screen; participant chooses avatar owl. (**B**) Participant tries to steer avatar on the star-studded path by appropriate movements of the target joint, while being instructed not to move the other joints or trunk. (**C**) An accommodation phase during which the participant was made familiar with steering the owl preceded the 30 s of measurement. The path was calibrated for each individual participant. Movements were performed within 90% of the active range of motion of each joint. The last 5 s were implemented to ensure that the participants continued playing until the end but were not analyzed. (**D**) Placement of sensors for the lower and upper extremities with indication of coordinate system. Sensors were attached with Velcro straps. To avoid that participants could steer the avatar with compensatory movements of the more proximal joints, we used master-slave sensor pairs for each joint to ensure that appropriate movements were used to steer the owl. The participants were seated on a pedestal or an adjustable chair for the lower and upper extremity testing, respectively.

In collaboration with the company Reha-Stim Medtech AG (previously YouRehab, Schlieren, Switzerland), we created a game-like assessment to measure SVMC with the name ‘Catch the stars’ (Fig. 1A,B). In this game, joint movements were recorded using accelerometer sensors. The signals were integrated online, enabling the participants to steer an avatar owl on a star-studded, predefined path by moving the target joint in an isolated manner. The path was designed to challenge the players within 90% (the exact path is depicted in Fig. 1C) of their individual active range of motion (which was calibrated beforehand) for 30 seconds.

The sensors were attached proximally and distally of each relevant joint (see Fig. 1D). The proximal sensors acted as reference for the more distal ones, preventing compensatory movements of a more proximal joint of having any beneficial influence on the flight path of the avatar. For example, a shoulder anteversion movement would not influence the path of the owl when it had to be steered with elbow flexion and extension movements because the difference between the sensors would remain the same. This was implemented because the influence of compensatory movements had been a problem in a previous study^34^.

While playing, the sensors not used for steering (see Fig. 1D) recorded any extraneous movements, which were then analyzed offline. Simultaneously, we videotaped the participants to verify the accelerometer signals. When playing with the lower extremities, the game was controlled by flexing and extending the individual hip, knee, or ankle joints. The participant was able to steer the avatar owl upward by hip flexion, knee extension or dorsal flexion of the ankle, respectively. On the contrary, hip extension, knee flexion and ankle plantar flexion resulted in a downward movement of the owl. For the upper extremities, the game was played by abducting and adducting the shoulder, flexing and extending the elbow, wrist, and fingers, and by pronating and supinating the lower arm.

### Participants

We investigated the SVMC of lower limb joints in 31 neurologically intact adults, 31 neurologically intact children, and 23 pediatric patients (all diagnosed with cerebral palsy). When assessing the SVMC of the upper extremities, 33 neurologically intact adults, 31 neurologically intact children, and 25 pediatric patients with various upper motor neuron lesion diagnoses participated. The participants’ characteristics can be seen in Table 1.

**Table 1:** Characteristics of participants for both the lower and upper extremity assessgame.

	Lower extremities			Upper extremities		
	NIA	NIC	P	NIA	NIC	P
Number of participants (females)	31 (15)	31 (16)	23 (9)	33 (15)	31 (16)	25 (9)
Age [y] (median; [1st, 3rd quartile])	33.9 [27.5, 38.4]	11.0 [8.7, 13.7]	10.6 [8.7, 15.3]	32.5 [27.9, 38.3]	11.6 [8.5, 13.9]	10.4 [8.8, 15.2]
Dominant side (left, right)	8, 23	3, 28	8, 15	6, 25	2, 29	7, 18
More affected side (left, right)	—	—	16, 7	—	—	17, 8
Diagnosis (CP, stroke, TBI)	—	—	23, 0, 0	—	—	17, 6, 2
GMFCS levels (1, 2, 3, 4, 5)	—	—	8, 4, 5, 6, 0	—	—	—
MACS levels (1, 2, 3, 4, 5)	—	—	—	—	—	3, 11, 10, 1, 0

Abbreviations: NIA = neurologically intact adults; NIC = neurologically intact children; P = pediatric patients; CP = cerebral palsy; TBI = traumatic brain injury; GMFCS = Gross Motor Function Classification System; MACS = Manual Ability Classification System.

### Summary of data analysis

**Figure 2:**
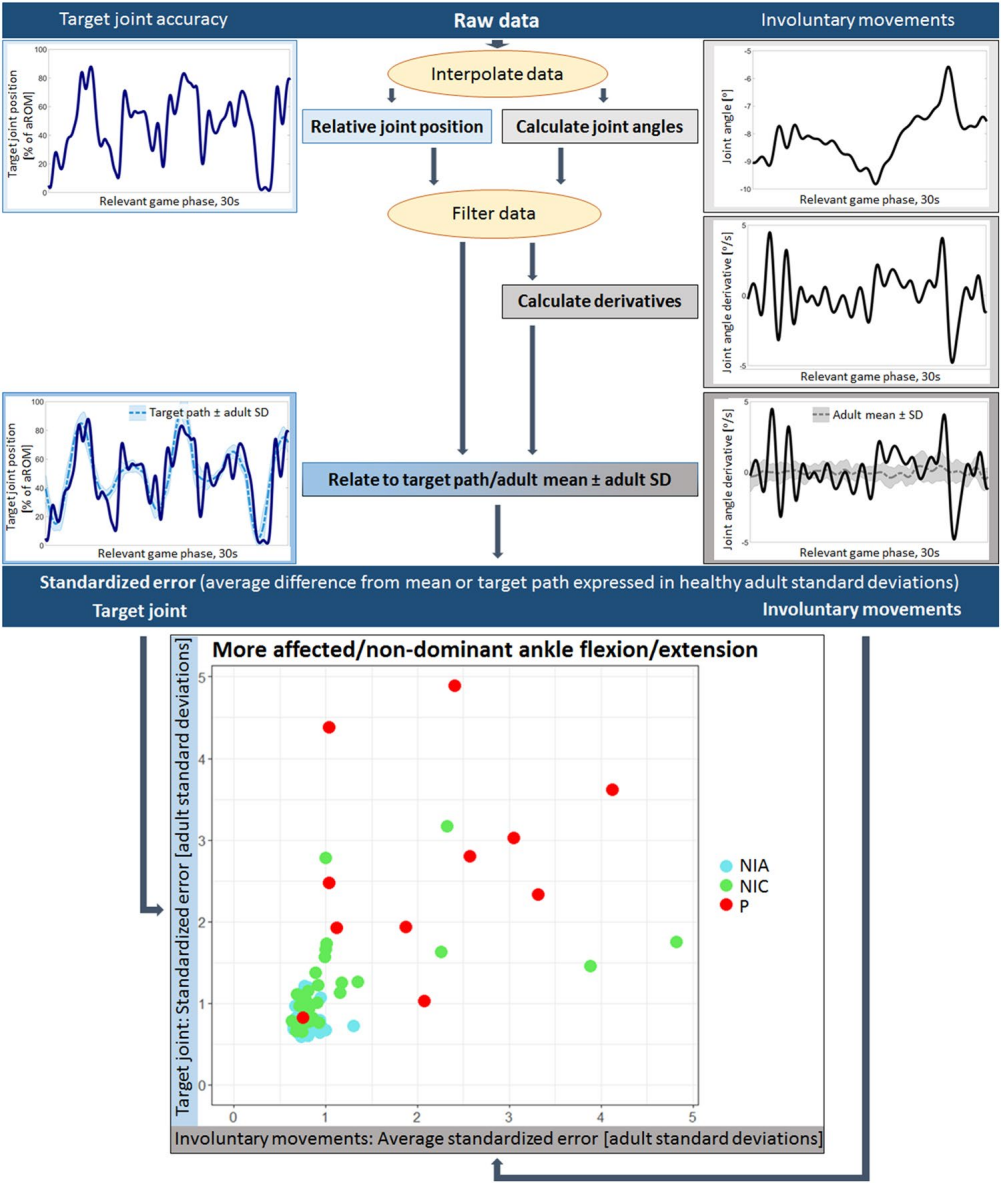
Summary of data analysis steps and visualization of outcome scores. The assessgame splits selective voluntary motor control (SVMC) into target joint accuracy and involuntary movements. We visualized the algorithm analyzing the raw accelerometer data resulting in standardized error scores for both outcome metrics. For the target joint, the numbers between 0 and 100 reflect the percentage joint position relative to the calibrated active range of motion. The involuntary movements were analyzed by first calculating the actual joint angle and then the derivative to quantify changes in position. This was done so that patients who were unable to maintain the starting position were not penalized. Finally, the standardized error expresses how many adult standard deviations the player was away from either the target path or the adult mean (involuntary movements) on average. The exact procedure is described in the supplementary material section. The scatterplot (described in more detail in Fig. 3) displays the accuracy metric on the y-axis and the averaged standardized errors of up to 11 other joints, accounting for the involuntary movement score on the x-axis. Abbreviations: aROM = active range of motion; SD = standard deviation; NIA = neurologically intact adults; NIC = neurologically intact children; P = pediatric patients.

We wrote an algorithm in MATLAB to calculate the metrics of target joint accuracy and involuntary movements from the accelerometer data (Fig. 2 summarizes the process for both metrics). A detailed description of the algorithm can be found in the supplementary material (starting with the subheading ‘Algorithm: Angle calculation’). In summary, for the accuracy score, the avatar position was calculated relative to the calibrated range of motion (percentage score). Because one sensor of the pair acts as reference for the other, shifts in the plane the game is played in do not influence the avatar position. After filtering the data, the relative avatar position and the reference adults were used to create the final score. The difference between the target path and the avatar positon was divided by the adult standard deviation around the target path. This allows to interpret deviations from the target path with respect to target path difficulty (a more difficult trajectory leads to larger standard deviations). Finally, all deviations were averaged into one accuracy score.

For the involuntary movement score, joint angles needed to be calculated first (the exact formula for each joint can be found in the supplemental material, subheadings ‘Algorithm for the lower/upper extremities’). After filtering, a time derivative of the joint angles was calculated. This means that changes in joint angles were analyzed and not the absolute position. Thus, patients were not penalized if they were not able to take up the predefined starting positions of the non-target joints (e.g. a 90-degrees angle of the elbow when playing the game with the shoulder). Finally, these derivatives were related to the reference adults, again by dividing the difference between joint position and ideal path by the adult standard deviations. However, instead of calculating the adult standard deviation around a zero line, indicating no movement, we used the mean of the adult movements, because some movements might be physiological. For example, flexing the knee while also flexing the hip joint is technically speaking an involuntary movement, but physiological because it shortens the lever arm and thus makes the movement easier. The standardized errors were then averaged per joint and thereafter all joints (consisting of up to eleven joints, depending on the target joint) were combined to one involuntary movement score.

In the end, one standardized error value for the target joint and one for the involuntary movements were used as outcomes for further analyses. The data of neurologically intact children were used to relate the performance of patients to age-matched peers, as some involuntary movements may occur also in neurologically intact children of younger age^35^.

If a participant was able to play with a joint but there were missing data for the involuntary movements due to failure of the sensors, or the accelerometer output did not match the video recordings, we imputed a value by used multiple imputation by chained equations (MICE)^36^. For further information on the exact procedure, we refer to the supplementary material.

**Figure 3:**
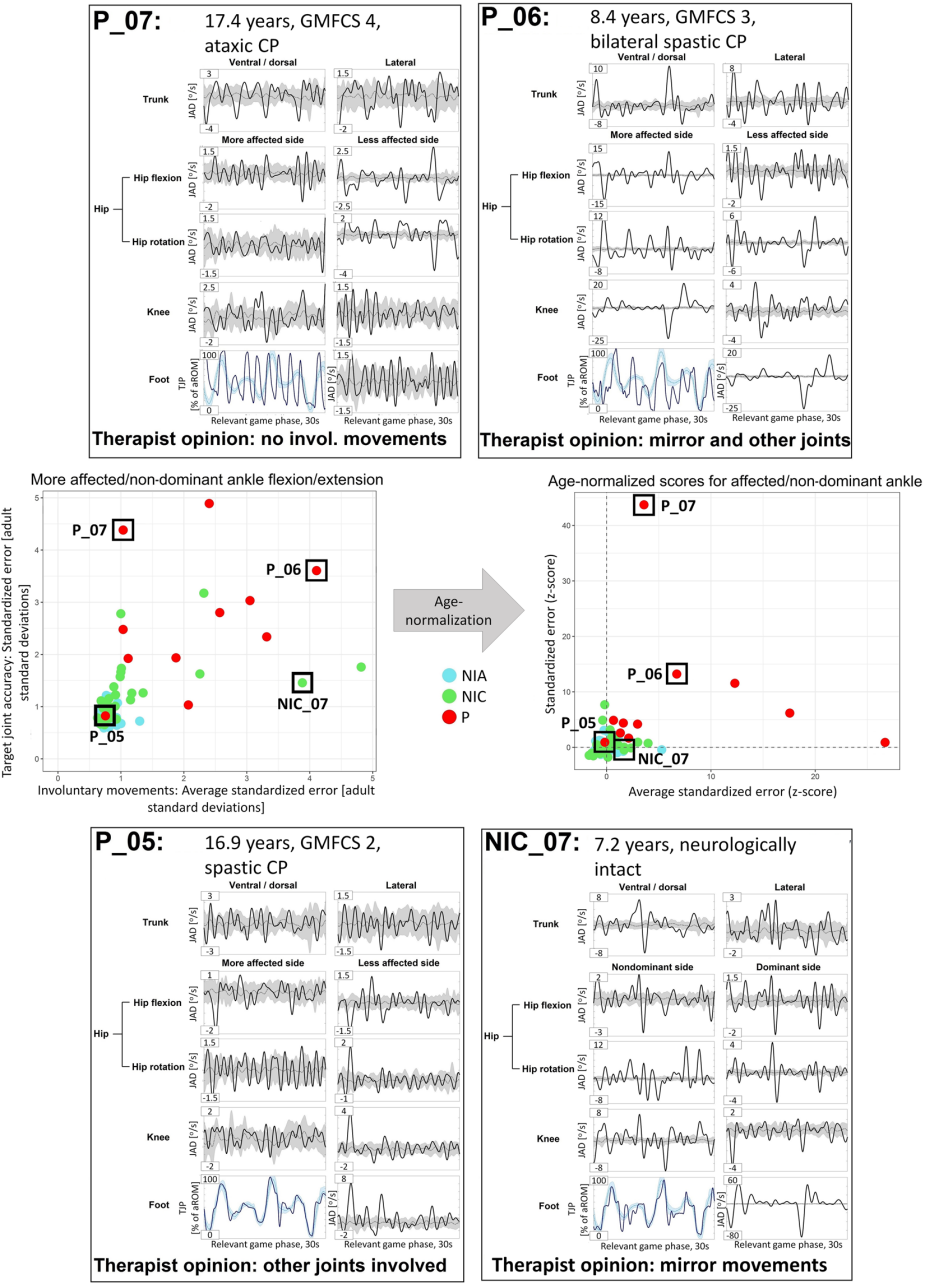
Examples of individual composition of outcome scores. More affected/non-dominant ankle flexion/extension as an example plot. The individual coordinates on the accuracy and involuntary movement plot are broken down into joint angle derivatives for every joint monitored during the assessgame play through. Therapist opinions are also provided. Age-normalizing was performed via z-transformation by creating peer groups ranging ± 1 year around the integer age of the current participant when looking at neurologically intact children and patients. The neurologically intact adults were not subjected to subgrouping. The resulting z-scores for each participant are presented in the age-normalized plot. Z-scores were constructed such that a positive value corresponds to a worse than average score and negative scores conversely indicate a better than average score, analogous to the non-age-normalized plot (larger values indicate worse scores). Abbreviations: aROM = active range of motion; TJP = target joint position; JAD = joint angle derivative; NIA = neurologically intact adults; NIC = neurologically intact children; P = pediatric patients; CP = cerebral palsy; GMFCS = Gross Motor Function Classification System.

Figure 3 displays the outcome plot of the affected (patients) or non-dominant (neurologically intact participants) ankle joint. Four participants have been marked and the composition of their scores is shown in individual graphs, analogous to the ones displayed in Fig. 2. They were chosen such that they exhibit all possible combinations of good/poor target joint accuracy and few/many involuntary movements. The score of the target joint accuracy (y-axis) is the mean standardized error value of the ankle movement displayed in blue (always in the bottom left corner, since the affected/non-dominant ankle of all the selected participants happened to be the left side). The score of the involuntary movements (x-axis) is the mean of the trunk (lateral and ventral averaged), hips (flexion and rotation averaged for both sides individually), knees, and the contralateral ankle. The following observations about target joint accuracy and involuntary movements can be made for the highlighted participants:

- Patient 07 (P_07): The accuracy error score is increased because the movement oscillates between the maximum and minimum of the active ROM, rarely staying on the target path. Involuntary movements are few, which is indicated by the bold line of the participant staying close to or within the boundaries of the reference adults. The involuntary movements that occur are mainly located in the contralateral hip joint, i.e. flexion as well as rotation.
- Patient 06 (P_06): For the target joint, the distance from the target path is larger predominantly in the less extreme joint positions (not near the limits of the active ROM), causing the elevated error score. However, the movement is performed with less error compared to participant 1. Involuntary movements are frequent and especially strong in the contralateral ankle joint (mirror movements), and ipsilateral hip and knee joints, which is also demonstrated by the fact that the reference bands around the mean of the neurologically intact adults are barely visible.
- Patient 05 (P_05): The target joint movement is always on point, with the exception of a small slip in the first part of the second half of the path. Noticeable, involuntary movements occur only in the very beginning of the game. They are, however, weak as indicated by the relatively low peaks in the curves. The low frequency and intensity lead to a small error value. Interesting here is that the therapist noted that other joints were involved while playing the assessgame.
- Neurologically intact child 07 (NIC_07): The target joint error was low, indicating good accuracy. The strongest involuntary movements occurred in form of mirror movements. Hip rotation on both sides, knee flexion/extension especially on the ipsilateral side, and ventral/dorsal trunk movements presented large amplitudes too, contributing to the increased error score.

After normalizing for age (z-transformation), the positions of P_07 and P_05 have not changed noticeably. In contrast however, NIC_07 who, due to the young age (7.2 years), is now closer to the lower left corner indicating good accuracy and control of involuntary movements when compared to peers. Please note that for each target joint, such analyses were made.

In a first effort to validate this approach, we determined the relationship between the assessgame outcomes accuracy and involuntary movements and several factors, which we considered relevant for estimating the level of SVMC. We therefore included the following factors in the models: (i) Routine clinical, ordinal-scaled SVMC assessments, namely the Selective Control Assessment of the Lower Extremity (SCALE)^27^ and the Selective Control of the Upper Extremity Scale (SCUES)^28^. (ii) Because the SCALE and SCUES scores of each joint combine the performance of the target joint and the occurrence of involuntary movements, the therapist additionally rated movement execution. This ‘therapist opinion’ categorized the movement as being either selective or any possible combination of involuntary movements occurring at the trunk, contralateral side (mirror movements), or other joints. This was then expressed as a sum score from 0 (selective) to 3 (all 3 involuntary movements occurring). (iii) Clinical classifications reflecting the severity of the disability evaluated by the Gross Motor Function Classification System (GMFCS)^37^ for the lower extremity and the Manual Ability Classification System (MACS)^38^ for the upper extremity. (iv) Finally, as SVMC develops over the lifespan, we included the age of the participant.

Since we considered each joint measurement individually for the analysis, linear mixed models were chosen to account for the fact that each participant contributed multiple outcome scores. The above-mentioned factors (i to iv) were entered as fixed effects in linear mixed models. Random effects in the model allowed us to account for by-subject and by-joint variation in the assessgame score.

**Figure 4:**
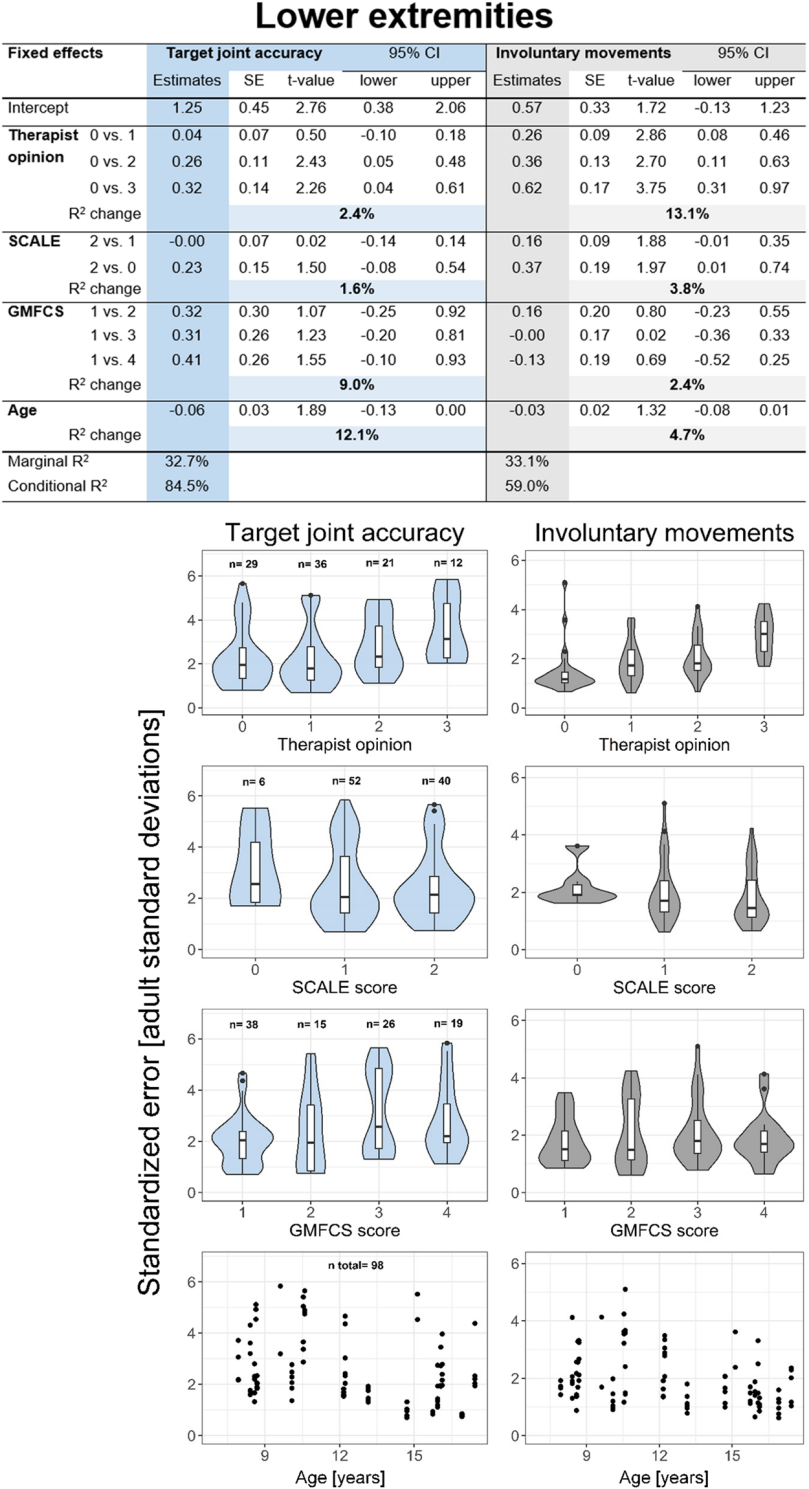
Linear mixed model results of the lower extremities. Linear mixed models and violin plots combined with box plots displaying the accuracy and involuntary movement outcomes of all lower extremity joints individually. Marginal R^2^ describes how much variance is accounted for by the fixed effects and conditional R^2^ by the total model. R^2^ change indicates how much the marginal R^2^ increased when adding the predictor to the model. An example of how to read the table (from left to right): Looking at therapist opinion, a change of predictor level from 0 to 3 yields a log-transformed outcome score increase of 0.32 (level 3 minus level 1), the standard error is 0.14 resulting in a t-value of 2.26 (this would be for a t-distribution). Since, however, for linear mixed models the underlying distribution is not known, we also added the bootstrapped confidence interval to better assess if a significance for a certain predictor could be established. Abbreviations: SE = standard error, CI = confidence interval, SCALE = Selective Control Assessment of the Lower Extremity, GMFCS = Gross Motor Function Classification System.

**Figure 5:**
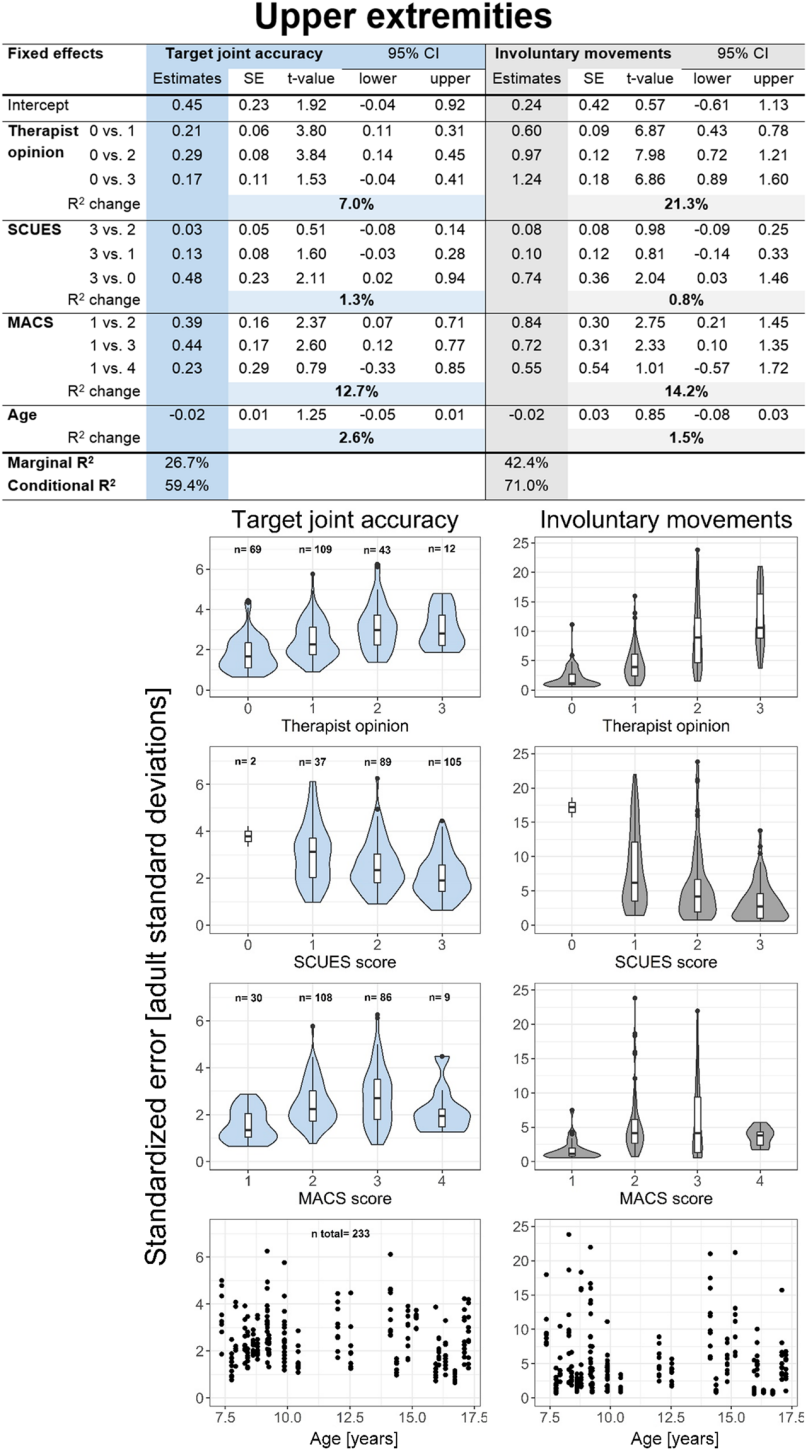
Linear mixed model results of the upper extremities. Linear mixed models and violin plots combined with box plots displaying the accuracy and involuntary movement outcomes of all upper extremity joints individually. Marginal R^2^ describes how much variance is accounted for by the fixed effects and conditional R^2^ by the total model. R^2^ change indicates how much the marginal R^2^ increased when adding the predictor to the model. Abbreviations: SE = standard error, CI = confidence interval, SCUES = Selective Control of the Upper Extremity, MACS = Manual Ability Classification System.

Figures 4 and 5 display the linear mixed models of the lower and upper extremity, respectively. The individual subplots below are violin and box plots indicating the relationship between the assessgame outcomes and the factors (independent variables) of the linear mixed models. The number displayed in the age subplots of the lower and upper extremities represents the number of total joint measurements that were available for the analysis. In addition to the exclusion of all non-playable joints, one measurement point of the upper extremities had to be excluded (dominant elbow of Patient 03) due to missing therapist opinion rating and lack of video recording required to assess selectivity of the movement retrospectively. All assumptions of the linear mixed models were met after log-transforming the dependent variables.

For the involuntary movements of both the lower and upper extremities, therapist opinion was the best predictor. Furthermore, with an increasing therapist score (more involuntary movements at various joints), the log-transformed score also increased. When considering the involuntary movements of the arms, the MACS level was a strong predictor as well. Changes in SCALE/SCUES, GMFCS (for the lower extremities) and age predicted the involuntary movement outcome score of the assessgame for both arms and legs less well.

The best predictor of the target joint accuracy score for the lower extremities was age, as indicated by the R^2^ change, followed by the GMFCS level. However, the t-value as well as the bootstrapped 95% confidence interval suggest both estimates were non-significant. The therapist opinion and SCALE were the weakest predictors. The marginal R^2^ value, indicative of the amount of variance explained by the fixed effects, is approximately the same for both the target joint accuracy and the involuntary movement score of the legs. The entire model (conditional R^2^), including random effects, could explain a substantially larger portion of variation for the accuracy score than the involuntary movements.

The best predictor of the target joint accuracy score of the upper extremities was the MACS level, followed by therapist opinion. The SCUES and age were the weakest predictors. The marginal R^2^ was larger for the involuntary movements score, meaning that the fixed effects could explain more of the variance of the involuntary movements. Overall, less variance could be explained for the joint accuracy score.

## Discussion

Clinical assessments evaluating selective voluntary motor control (SVMC) are either subjective tests measured on an ordinal scale or require expensive equipment. The alternative presented in this manuscript seeks to solve both problems by using relatively inexpensive accelerometers to quantify objectively the accuracy of the target joint as well as the occurrence of involuntary movements. The interpretation of both scores was shown exemplary in the results section. Finally, we used linear mixed models to estimate the importance of different predictors of the assessgame outcome scores. Therapist opinion, measuring if the movements were done selectively or not, was found to be the strongest one for both lower and upper extremities when predicting the involuntary movement score.

The largest difference between the assessgame and other tools measuring SVMC is that the assessgame can divide the performance into two components, one concerning the accuracy of the target joint movements and one evaluating the involuntary movements. This is possible because the assessgame uses a predefined path, which players needed to stay on. This, in our eyes, perfectly provides the “*demands of a voluntary posture or movement”* defined by Sanger *et al*.^1^. In the clinically used assessments, such as the SCALE and SCUES, the therapist provides acoustic cues, which the participant tries to follow. Later, at least for the SCALE, the therapist consults the video recordings to estimate how timely the movements were performed and deducts points if they were executed too slow^27,28^. Thus, a compound score is created, combining target joint and involuntary movements. The same is true for the laboratory-based measures using surface electromyography (sEMG)^33,39^, which also create a compound score. The optimal solution would be to combine the predefined path with the sEMG analysis to fulfil the definition of SVMC completely (i.e., making the score less dependent on muscle strength, which is a requirement to overcome gravity and could mask the ability to selectively activate muscles).

Furthermore, playing the assessgame is a more difficult task compared to the routine clinical assessments and, therefore, may elicit more involuntary movements. Studies found that attentional demands^40^ and task difficulty^41^ negatively affect the inhibition of involuntary movements. Besides the fact that our assessgame provides a much more challenging task than current clinical tools, there is also a shift in attention. During the SCALE/SCUES, the participants fully focus on the movement and are encouraged to observe the limb performing the movement. For that reason, participants perform the SCALE with an elevated ankle joint. Although we purposely used the same testing position for the assessgame, the participants focused on the screen for receiving direct feedback on the accuracy part of their performance. This leaves only peripheral vision as a possibility for visual control of limb movements, which might have influenced task performance especially of patients with reduced proprioception.

Moreover, the discrepancy between passive and active ROM for the various SVMC measures needs to be addressed. According to the definition of SVMC, the sEMG laboratory-based method is considered the best option because it actually analyzes muscle activation patterns, even in the absence of movement^33^. The SCALE and SCUES both evaluate the participant’s target joint performance in relation to the passive ROM. If the participant does not reach 50% (SCALE) or even 85% (SCUES) of the passive ROM, points are deducted. For our assessgame, we decided to calibrate the game with the active ROM of the participant for every round. We argue that this is an advantage because it means the assessment is affected less by strength deficiencies and the outcome score may better reflect a true reduction of SVMC. This provides relevant therapeutic information, because it could indicate at which joint training may be most beneficial for the patient. For example, it is conceivable that patients with strength deficiencies get a worse score when performing a clinical assessment because they cannot reach the ROM requirements, despite them being capable of selectively activating the required muscles in a certain pattern. In such a case, relative straightforward strength training may yield the desired improvements without the need to undergo a more difficult coordinative training where the patient should simultaneously try to inhibit undesired involuntary movements actively.

Finally, one of the main reasons for this kind of interval-scaled assessment is the supposedly greater sensitivity. When raters observe involuntary movements occurring in the SCALE/SCUES, the score will be submaximal, regardless of how prominent the involuntary movements are. This is observable when looking at the graphs plotting the assessgame outcomes against the SCALE/SCUES, especially for the upper extremities. When the joint is classified with a better SCALE/SCUES score, the median of the assessgame error values tends to be lower and more scores seem to lie in lower ranges as indicated by the density plots. Albeit, there is still a large overlap. Of course, it is difficult to make the grading much finer for these assessments without sacrificing simplicity. One possibility to address this issue would be by looking at the frequency as well as the prominence of involuntary movements, as this is done in the Zurich Neuromotor Assessment 2 (ZNA2)^42^. In fact, the assessgame does this with an even finer grading. As can be seen in Fig. 3 when looking at P_05, the therapist noted involuntary movements occurring in other joints during ankle flexion extension. Involuntary movements occurred mainly in the contralateral side during the onset of the game. They were, however, low in amplitude and did not occur subsequently, thus the participant also got a low score for the involuntary movements, placing the displayed level of SVMC amongst those of neurologically intact adults.

Concerning the results of the linear mixed models, therapist opinion was the strongest predictor for the involuntary movements of the assessgame and generally speaking, more predictive of the involuntary movement score than the accuracy score. This coincides with the expectations, since the therapist was instructed to note the involuntary movements occurring and not look at the accuracy of the movement. Moreover, the assessgame outcome scores change as expected when there is a change in predictor level. Future studies might use assessments with a scoring system comparable to the ZNA2 tool and assess how strong a predictor with finer grading of involuntary movements is for this kind of outcome measures.

Similar to the therapist opinion score, the SCALE and SCUES focus more on the involuntary movement part of the movement (mirror and other joint movements as well as trunk movements) than the actual movement, where the main criterion is movement amplitude. Hence, one would expect that these tools are better predictors for the involuntary movements score. This is observable for the lower but not for the upper extremities. However, the number of joint measurements for the lowest categories (indicating no selective voluntary movement possible) of both the SCALE and SCUES was very small, as would be expected.

Concerning the lower extremities, changes in severity of disability were found to have little to no influence on the involuntary movement score, contrary to what can be observed for the target joint accuracy (without changes in level yielding significant differences). For the upper extremities, a change from the reference category (MACS level 1) to other levels was found to yield significant differences in scores, at least for comparisons with levels 2 and 3. Yet again, it has to be noted that we had very few joint measures for MACS level 4. Indeed, only one patient with such a MACS level participated in this study. Noteworthy is the pattern that can be seen, which is that for an increasing MACS level the involuntary movement scores decline. A possible explanation for this might be that with increasing severity of disability, the involuntary movements occur with less amplitude.

One of the main considerations to be addressed is the time to administer the assessgame in its current form. Even though especially therapist opinion was found to be a strong predictor of the assessment game outcomes, other factors may also have an influence on the outcome score. For example, the duration of this assessment is approximately 20–30 minutes for the lower and 30–40 minutes for the upper extremities. Naturally, motivation and concentration may play a larger role than during the SCALE/SCUES, which takes no more than 15 minutes to perform with the participants^27^. This could be a reason why the clinical assessments SCALE and SCUES entered into the linear mixed models were not as strong a predictor as one could have expected. One possibility to increase the feasibility of the assessgame would be to use it in its current form only for pre-assessment, thereby identifying the most relevant impairments in movement accuracy and involuntary movements. After applying an intervention, solely the identified joints would be reevaluated. In addition, the number of sensors could be reduced in future adaptations of the assessgame to make it easier for therapists to handle.

Furthermore, the number of joint measurements was relatively small when considering the number of predictors added to the model. Particularly for the lower extremities, where only 98 joint movements were performed out of 144 possible ones. This was mostly due to the fact that playing the game with the ankle joints was difficult for patients, because the minimally required active ROM (approximately 20 degrees) was not reached.

A final consideration are the accelerations due to movement acting on the sensors in addition to the earth’s gravity. We argue that these additional accelerations are relatively small in comparison (see methods section) to gravity and mathematically accounted for by using the adults as reference group. Nevertheless, they are present and will slightly impair the precision of the angle calculation.

Concluding, we could show that this method seems promising in quantifying SVMC even in pediatric patients with upper motor neuron lesion, who show more challenges when participating in clinical assessments compared to adult patients. Consecutive psychometric studies are needed to determine, especially, the reliability and responsiveness.

## Materials and Methods

### Participants

The occurrence of involuntary movements can be physiological early and later in life^43^. For example, in neurologically intact children, the occurrence of mirror movements of the upper limbs is physiological up to an age of about 10 years^35^. But also in adults aged 50–80 years, a submaximal finger pressing task resulted in increased contralateral finger pressing (mirror movements) compared to young adults (10–30 years)^44^. These observations demonstrate the need to collect reference values in healthy persons (both young adults as well as age-matched children) to interpret findings in patients with neurological lesions correctly.

We, therefore, aimed to recruit 30 neurologically intact adults, aged 18 to 50 years, to obtain reference values. Participants were recruited by convenience sampling at the Rehabilitation Center Affoltern am Albis (Switzerland).

Furthermore, we aimed to recruit 30 neurologically intact children, aged 6–18 years, to interpret results in children and adolescents with upper motor neuron lesions correctly. This sample was recruited by quota sampling to have an appropriate age distribution in the group. Since the occurrence of involuntary movements has been more frequently reported for the younger age categories^35,45,46^, the goal was to enroll more participants in the age range of 6 to 10 years than in the range of 10 to 18 years, because of the expected larger variability in our assessgame in the younger age group.

Participants within the patient sample had to have a diagnosed upper motor neuron lesion, the ability to understand und follow simple instructions, an age between 6 and 18 years, and the ability to sit upright (with backrest support) for up to 1 hour (because the assessment game was part of a more comprehensive test battery). Exclusion criteria were surgical interventions or Botulinum toxin treatment in the past 6 months. In- and outpatients of the Rehabilitation Center Affoltern am Albis were recruited, again by convenience sampling.

To describe the healthy reference population, we used the characteristics age, gender, and dominant side of the upper and lower extremities. Side dominance of the upper extremities was evaluated by asking the participant which hand they used to write and draw. If the participant was unsure, pen and paper were provided and they were asked to write their name. The hand used to hold the pen was declared the dominant side. Analogous, dominance for the lower extremities was established by asking the participant with which foot they preferred to kick a ball. If the participant was unsure, a football was provided. The foot that was used to kick the ball was noted as the dominant foot. For patients, we additionally recorded the diagnosis, the more affected side (either the side indicated by the physiotherapist or occupational therapist or, if both sides were equally affected, the non-dominant side), and the MACS and GMFCS levels. The MACS classifies how children with cerebral palsy handle objects in daily activities. Level 1 means that the child can handle objects easily and successfully whereas children at level 5 do not handle objects at all^38^. The GMFCS standardizes the classification of gross motor function, emphasizing trunk control and walking ability of children affected by cerebral palsy^37^. Children at GMFCS level 1 perform all the activities neurologically intact children of the same age can, allowing for slight limitations in speed and quality of movements. Children with GMFCS level 5 exhibit difficulties in head and trunk control in most positions or achieving any voluntary control of movement at all^47^.

The ethical committee of the canton of Zurich, Switzerland (PB_2016_01843) approved this study. Either the participant and/or the legal guardian gave written informed consent. All methods were in accordance with the necessary guidelines.

### Assessgame technology

Reha-Stim Medtech AG used wireless Shimmer sensors for their wearable master/slave kinematics capture system. The master unit consisted of three 3-axis accelerometers, one 3-axis gyroscope, two 3-axis magnetometers. The slave unit consisted of two 3-axis accelerometers, one 3-axis gyroscope, and two 3-axis magnetometers. The sampling frequency was on average slightly below 40 Hz. It was either 29 Hz (approximately 70% of the time) or 58 Hz (roughly 30% of the time). This was due to the fact that the sensor transmitted the position readings only if all sensors had a reading. The company provided us with the accelerometer output for each master and slave sensor, which we analyzed offline.

Sensor accuracy and precision (repeatability, defined as standard deviation of measurement) were tested by mounting a sensor and an analog inclinometer to a hinge joint, which could be locked in place. Thereafter, we varied the angle of the hinge joint between 0 (sensor y-axis parallel to the gravity vector) and 90 degrees (sensor y-axis perpendicular to the gravity vector) stopping every 5 degrees and measuring for 10 seconds. We repeated this procedure 5 times. Sensor accuracy was within 1.9 degrees on average (range 0.5 to 3.2 degrees) and precision within 0.16 on average (range 0.12 to 0.20 degrees).

Furthermore, we tested sensor accuracy and precision when in motion. This was done by mounting two sensors on a rigid 90-degree angle 30 cm away from the center of rotation and playing the game with a more proximal joint, comparable to an adult (height approximately 1.8 m) playing the game by abduction of the shoulder while holding the elbow perfectly still. For this condition, the accuracy was 0.7 degrees and the precision 1.3 degrees. This can vary depending on arm length and movement speed. This indicates that additional accelerations due to movements of more proximal joints have a relatively small impact (within 1 and 3%, depending on the tested joint) compared to the earth’s gravitational field, when movements are within the range of the games target path.

### Setup and procedure for assessing the lower extremities

Participants played the game while sitting on a pedestal with their legs hanging freely. The only exception was the ankle joint, in which case the shank was placed on a support beam (only the target side), so that the participant was able to see the foot while playing (analogous to the SCALE^27^, where the shank is supported by the therapist’s hand). The sensors were attached to the participants’ upper thigh, lower thigh, upper shank, lower shank, and foot with Velcro straps (Fig. 1D).

During the 10 seconds calibration, the active range of motion was established for each target joint. The active range of motion was chosen over the passive one, to better distinguish between SVMC and strength deficiencies. To ensure that playing the game over time would not become too strenuous, assessors made sure that ranges of motion for the hip, knee, and ankle did not surpass approximately 45, 90, and 90 degrees, respectively, and 5 degrees of the range were pruned off the top and bottom. During the accommodation phase, participants were asked if the owl responded adequately to the movements and if questionable, the participant was asked to position the avatar at the top and bottom of the screen. If this was not possible, the game was recalibrated.

As we had noticed in pilot trials that maximal hip extension was difficult to achieve when playing, the calibration was adapted by laying a folded towel beneath the thigh. To mitigate a possible learning effect and to get used to the steering mechanism, the participants were familiarized to play the game with 3 of the 6 joints before the actual measurements took place. Participants could choose if they preferred to practice with the right or left side. After these trial rounds, the participants played all joints on both sides in a randomized order to account for any learning effect. For every playing round, the therapist documented if the target movement was performed without occurrence of any involuntary movements or not. If involuntary movements were visible, the therapist noted if they occurred at the same but contralateral joint (mirror movements), at any other joints where sensors were attached, and/or the trunk. Any combination or all of the involuntary movements could be noted. Additionally, before playing the assessgame, we evaluated the joints of the lower limbs using the German version of the SCALE^48^.

### Setup and procedure for assessing the upper extremities

For the upper extremities, the game was played by abducting and adducting the shoulder, flexing and extending the elbow, wrist, and fingers, and by pronating and supinating the lower arm. Participants sat on an adjustable chair, with the hip, knees, and ankles in a 90-degree angle and the lower arms resting on the table such that the elbow was in a 90-degree angle. The sensor attachment is displayed in Fig. 1. Sensors were placed in a manner that, when optimally moving, the y-axis moved in the desired movement plane. The Velcro attachment for the elbow slave sensor was positioned as close to the cubital crease as possible, thus preventing excessive movement when pro-/supinating the forearm. When the participant played with the shoulder and elbow joint, the arms were hanging, when playing with the lower arm, wrist or fingers, the lower arm was placed on a foam padding such that these movements were not restricted by the support of the arm.

The calibration procedure was done similarly as for the lower extremity, with the exception of the maximally allowed joint movements for the shoulder, elbow, lower arm pro-/supination, wrist, which should not exceed 70, 110, 135, and 135 degrees, respectively.

Participants played three trial rounds to familiarize with the assessgame using the following joints: fingers, forearm, and either elbow or shoulder. The fingers were chosen to familiarize the participant with the cyber-gloves. The participants also practiced the forearm movements, because pilot tests had revealed that pro- and supination were less intuitive movements to control the game. For the third trial round, the participants could decide if they either preferred playing with the elbow or the shoulder joint. Once acquainted with the different ways of steering, the participant played all 10 joints in a randomized order, to account for learning effects. The occurrence of involuntary movements was characterized the same way it was done for the lower extremities. Again, before playing the assessgame, we evaluated the joints of the upper extremities with a clinical tool to measure SVMC, the German version of the SCUES.

### Statistical analysis

The statistical analyses were performed in R^49^ (Packages: ‘ggplot2’^50^, ‘lme4’^51^, and ‘MuMIn’^52^) and done considering dominant/non-dominant side for neurologically intact individuals and less/more affected side for patients.

We used linear mixed models, more specifically the ‘lmer’ function from the package ‘lme4’, to better gauge the influence of the measures of SVMC (i.e. therapist opinion as well as SCUES and SCALE for upper and lower extremities, respectively), severity of disability, and age on the accuracy and involuntary movement error values of the assessgame in patients. Linear mixed model analysis allows to account for repeated measurements (one participant contributed multiple measurements) and thus assess the relevance of SVMC for all our assessgame outcomes individually^53,54^ Predictors of linear mixed models are either fixed effects, typically the model predictors of interest, or random effects. For the variables that were categorical (all except for age), we chose the best possible score as reference category for comparisons. Random effects, allowing us to account for by-subject and by-joint variation, can be set either for the intercepts, the slopes, or both. We decided to add only random intercepts in this study, so as not to over fit the model with too many predictors. By-joint variation was added to the model as nested predictor (more/less affected arm [2 levels] by tested joints per arm [5 levels]) or as individual joints (10 levels) if nesting was not possible due to overfitting. Target joints that were not playable were excluded from the analysis. We reported the coefficient estimates, standard errors (SE), t-values, and upper and lower bound of the bootstrapped estimate confidence intervals (CI). Approximations for explained variance by the fixed effects (marginal R^2^) and total model (conditional R^2^) were calculated using the package ‘MuMIn’.

## Supplementary information

Supplementary Information

## Data Availability

After a discussion with the ethics committee, we decided that due to the small number of patients in our rehabilitation center and the heterogeneity of the study group we can only provide the data upon reasonable request. For further information, please contact the corresponding author.
