# Supplementary material for: First validation of a novel assessgame quantifying selective voluntary motor control in children with upper motor neuron lesions
Source: Sci Rep. 2019 Dec 30;9:19972. doi: 10.1038/s41598-019-56495-8 (PMC6937339; doi:10.1038/s41598-019-56495-8)
Supplement: Supplementary file 1 — Supplementary Information [file 41598_2019_56495_MOESM1_ESM.pdf]

First validation of a novel assessgame quantifying selective voluntary motor control in children with upper motor neuron lesions

Jeffrey W. Keller[1,2,3,*], Julia Balzer[1,2,4], Annina Fahr[1,2], Jan Lieber[1,2], Urs Keller[1,2], Hubertus J.A. van Hedel[1,2]

Affiliations

[1] Rehabilitation Center for Children and Adolescents, University Children's Hospital Zurich, Affoltern am Albis, Switzerland

[2] Children's Research Center, University Children's Hospital Zurich, Zurich, Switzerland

[3] Doctoral Program Clinical Science, Faculty of Medicine, University of Zurich, Zurich, Switzerland

[4] Centre for Health, Activity and Rehabilitation Research, Queen Margaret University, Edinburgh, Scotland

*Corresponding author: jeffrey.keller@kispi.uzh.ch

Supplementary materials

Algorithm: Angle calculation

The rotation around the joint axis of interest (Sensor axis 3) is calculated using the arctangent function (see Fig. S1 for an example):

$$\alpha = arctangent\left(\frac{Sensor\ axis\ 1}{|Sensor\ axis\ 2| + constant}\right) \quad (1)$$

Absolute IMU values were used in the denominator to ensure a continuous function making case distinctions superfluous. Furthermore, a constant (10^{-17}) was added to every denominator preventing it from being zero. In the following step-by-step explanation of the algorithms we specify for every joint which values were used by indicating first the joint, then if master or slave, and finally which axes were used.

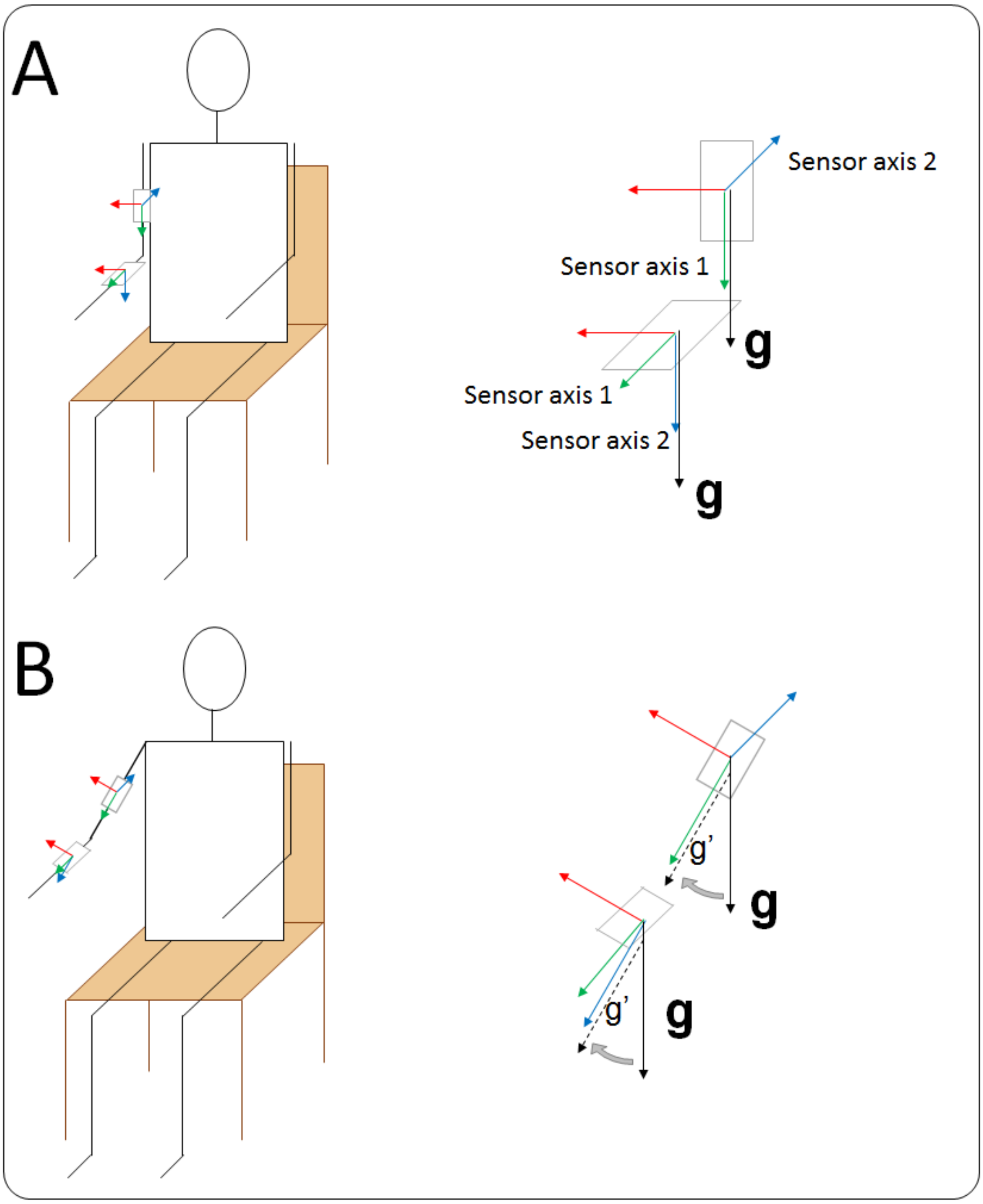

Fig. S1: Influence on the joint angle calculation by moving a more proximal joint. Exemplary calculation of the elbow angle (see formula (1)) when the shoulder is abducted. (A) Calculation of the elbow flexion angle of 90 degrees using sensor axes 1 and 2 while the shoulder abduction angle is 0 degrees. (B) By projecting the gravitational vector (g → g') on the plane described by sensor axis 1 and 2 the influence of the shoulder abduction can be eliminated, thus correctly calculating the elbow flexion angle of 90 degrees.

Abbreviations: g= gravitational vector; g'= projection of gravitational vector

Algorithm for the lower extremities

We used the following steps in the algorithm to calculate the joint angles and thereafter the 'standardized error values' (see Fig. 2) for the target joint accuracy and involuntary movements of the lower extremities:

1. Cut raw data to relevant 30s-phase leaving out the accommodation (25s) and end (5s) phase.
2. Interpolate IMU output to a constant frequency of 66Hz homogenizing time between data points to 0.015s for later calculation of joint angle derivative of time and filtering.
3. Calculate lower extremity joint angles by calculating the arctangent (formula (1)) of the specified axes ratios (M=master, S=slave sensor, x/y/z= axes, see Fig.1 of the main text for axes labeling):
    a. Target joint angle relative to calibrated range of motion: Calculated online for avatar position and thus already in raw data.
    b. Trunk, always contralateral of target joint: Lateral (hip Mx/My) and ventral (hip Mz/My) later averaged to one score contributing equally.
    c. Hip, distinction made between the hip being target joint and not, because hip slave sensor attached to upper thigh (might lead to false rotation angle when hip flexed):
        i. If hip not target joint: Hip flexion (hip Sy/Sz), hip rotation (hip Sx/Sz) later averaged to one score contributing equally.
        ii. If hip target joint: Hip flexion relative joint position, hip rotation (knee Mx/Mz) separate involuntary movement score.
    d. Knee extension: Knee My/Mz – knee Sy/Sz
    e. Foot dorsal extension: Foot My/Mz – foot Sy/Sz
4. Filter with 6th order Butterworth zero-phase low-pass filter with a cutoff frequency of 1.5Hz (normalized cutoff frequency of 0.045).
5. Calculate time derivative of joint angles ensuring that movement is penalized not starting position.
6. Repeat steps 1 through 5 for every participant.

7. Visual inspection of outliers and removal of data points that do not coincide with videotaped movements.
8. Calculate difference from target path (for the target joint accuracy) and ideal path (mean of adults, for the involuntary movements) and divide by neurologically intact adult standard deviations around these measures, resulting in standardized error value. Average all values for one joint. If analyzed player is adult, she/he does not contribute to reverence group. Mean of the healthy adults as reference and not the theoretically possible zero movement, because some movements may be physiologically desirable (e.g. slight knee flexion while flexing the hip in a sitting position, to shorten the lever arm).
9. Average individual scores of involuntary movements to overall score: trunk (average of lateral and ventral movement), hip (either average of flexion and rotation or only rotation), knee extension, and foot plantar extension all contribute equally.
10. Repeat steps 8 and 9 for every participant.

Algorithm for the upper extremities

1. Cut raw data to relevant 30s-phase leaving out the accommodation (25s) and end (5s) phase.
2. Interpolate IMU output to a constant frequency of 66Hz homogenizing time between data points to 0.015s for later calculation of joint angle derivative of time and filtering.
3. Calculate joint angle of lower extremity done by calculating the arctangent (formula (1)) of the specified axes ratios (M=master, S=slave sensor, x/y/z= axes, see Fig.1 of the main text for axes labeling):
    a. Target joint angle relative to calibrated range of motion: Calculated online for avatar position and thus already in raw data.
    b. Trunk, always contralateral of target joint: Lateral (shoulder My/Mz) and ventral (shoulder Mx/Mz) later averaged to one score.
    c. Shoulder abduction (Shoulder My/Mz – shoulder Sy/Sz) and rotation (shoulder Mz/Mx – shoulder Sz/Sx): If shoulder is target joint only shoulder rotation score used, else both abduction and rotation averaged (see point 10 for contribution).

Rotation may cause unstable angle when slave sensor y-axis vertical to gravity vector, thus a 20 degree abduction needed for reliable calculation.

   d. Elbow flexion: Elbow My/Mz – elbow Sy/Sz. When for either sensor the y- and z-axis are perpendicular to gravity (±20 degrees) or both sensors' y-axes are perpendicular to gravity (±20 degrees), the measurement is invalid (stored as NaN).
   e. Lower arm pro-/supination: Elbow Sz/Sx – Hand sensor z/x. When for either sensor the y-axis is parallel (±20 degrees), the measurement is invalid.
   f. Wrist flexion: Elbow Sy/Sz – Hand sensor y/z. When for the elbow sensor the y-axis and the z-axis are perpendicular to the gravity vector (±20 degrees), a three axes solution is chosen: Elbow Sy/(Sz+Sx). If for the hand sensor the z- and y-axis are perpendicular to the gravity vector (±20 degrees), the measurement is invalid.
   g. Finger flexion: data from bend sensor can be used without further calculations.

4. Extract invalid measurements from joint angle calculations.
5. Filter with 6th order Butterworth zero-phase low-pass filter with a cutoff frequency of 1.5Hz (normalized cutoff frequency of 0.045).
6. Calculate time derivative of joint angles ensuring that movement is penalized not starting position.
7. Insert the extracted invalid measurements. This ensures correct position of valid measurements.
8. Repeat steps 1 through 7 for every participant.
9. Visual inspection of outliers and removal of data points that do not coincide with videotaped movements.
10. Calculate difference from target path (for the target joint accuracy) and ideal path (for the involuntary movements) and divide by neurologically intact adult standard deviations around these measures, resulting in standardized error value only if 20 reference values present. Average all values over time for one joint. If analyzed player is adult, she/he does not contribute to reverence group.

11. Average individual scores of involuntary movements to overall score: trunk (average of lateral and ventral movement), shoulder (either average of abduction and rotation [ratio of 1:(1-proportion of invalid measurements)] or only rotation), elbow flexion, lower arm pro-/supination, wrist flexion, and finger flexion all contribute equally.
12. Repeat steps 10 and 11 for every participant.

Multiple imputation by chained equations (MICE)

As mentioned in the main text, multiple imputation by chained equations (MICE) *(36)* were used to impute missing data due to sensor failure. The decision to do so was based on the fact that the score of the involuntary movements is an average across all the joints not used for playing the assessgame and the danger of over- or underestimating this average score if certain key joints went missing. Since, in this case, the data could be assumed to be missing at random, MICE offered a great opportunity to minimize bias.

Another possible source of missing data was exclusion due to unreliable sensor readings that led to false standardized errors. All joint angle derivative curves were evaluated and outlier curves were compared to the videotaped performance. If they did not coincide, the data point was also imputed.

Analyzing the lower extremities, 4920 'involuntary movements' were recorded, of which 39 had to be excluded due to sensor failure (0.8%), however, none were excluded because of unstable sensor readings. For the upper extremities, 11'336 'involuntary movements' were recorded, of these 135 were imputed due to sensor failure (1.2%), 34 because of unstable sensor readings (0.3%).

We used the following procedure to impute missing data points with MICE:

1. Data points of neurologically intact adults, children, and patients imputed separately.
2. Exclusion of participant if the target joint was missing.
3. Find location of the missing joint data.

4. Create new data set with missing data point, ipsilateral target joint, contralateral joint (mirror of missing), age, more affected side (or non-dominant side), gender, and contralateral target joint. Imputation was performed with the following predictors:
    a. ipsilateral missing joint = ipsilateral target joint + contralateral joint + age + more affected side + gender
    b. if contralateral joint also missing = missing data point + age + more affected side + gender + contralateral target joint
    c. if contralateral target joint missing = ipsilateral target joint + contralateral joint + age + more affected side + gender
    d. The variables age, more affected side, gender, and ipsilateral target joint can never be missing and thus do not have to be imputed
5. Run MICE procedure 50 times and impute the missing data point with the mean of the 50 imputed points.
6. Repeat steps 1 through 5 until there are no missing data points.

Algorithm of the lower extremity: Matlab code

```
% This algorithm uses data from accelerometers to approximate joint angles
% which occur as involuntary movements as part a target tracking task.

clear all; close all;
debug_out=0;

% Don't forget to change JOINT depending on whether TARjoint is on left or
right side
JOINT = 1 %HipR=1, HipL=2, KneeR=3, KneeL=4, FootR=5, FootL=6
if  mod(JOINT,2)== 1 %Required for switching of trunk movement sensor
    SIDE = 1 %odd number indicating that TAR joint is on right side
else
    SIDE = 0 %even number indicating that TAR joint is on left side
end
%When played with hip, other sensor chosen to approximate hip ratation
% because 'hip slave' attached to fleshy part of thigh
if JOINT == 1 | JOINT == 2
    HIP = 1
else
    HIP = 0
end

REF_HA = 31 %Number of reference adults present

%time inbetween 2 measurments after interpolating
Time_step = 0.015;%Chosen so that no information is lost because shortest
interval in output files 0.017s
fc = 1.5; fs = 1/Time_step; %frequency of measurments

%Definition of the lowpass filter
[b,a] = butter(6,fc/(fs/2),'low');

%Files to be analyzed
Choose_index =
{'01','02','03','04','05','06','07','08','09','10','11','12','13','14','15'
,'16','17','18','19','20','21','22','23','24','25','26','27','28','29','30'
,'31',
'C01','C02','C03','C04','C05','C06','C07','C08','C09','C10','C11','C12','C1
3','C14','C15','C16','C17','C18','C19','C20','C21','C22','C23','C24','C25',
'C26','C27','C28','C29','C30','C31','C32',
'P01','P02','P03','P04','P05','P06','P07','P08','P09','P10','P11','P12','P1
3','P14','P15','P16','P17','P18','P19','P20','P21','P22','P23','P24',
'P01R','P02R','P03R','P04R','P05R','P06R','P07R','P08R','P09R','P10R','P11R
','P12R','P13R','P14R','P15R','P16R','P17R','P18R','P19R','P20R','P21R','P2
2R','P23R','P24R'};

for subject_step=1:length(Choose_index)

    info1=dir('*.csv');
    for i=1:size(info1,1)
        filename=info1(i).name;
        if
strcmp(filename(1:length(Choose_index{subject_step})),Choose_index{subject_
step});
            break;
        end
        if i==size(info1,1)
            error('No file found')
        end
    end
```

```
    fid = fopen(filename,'r');
    C = textscan(fid, repmat('%s',1,48), 'delimiter',';',
'CollectOutput',true);
    fclose(fid);

    %Read .csv file to predefined length of the file
    matrix=[];
    matrix= dlmread(filename,';',[1 0 size(C{1},1)-2 47]);

    %Define target and actually flown (value) path
    target = matrix(:,3);
    value = matrix(:,2);

    %Cutting target vector to find relevant phase - start of game
    stopcondition=0;
    for i=2:length(target)
        if target(i)~=target(i-1) && stopcondition==0
            index_start=i;
            stopcondition=1;
        end
    end

    %Cutting target vector to find relevant phase - end of game
    stopcondition=0;
    for i=length(target):-1:2
        if target(i)~=target(i-1) && stopcondition==0
            index_end=i;
            stopcondition=1;
        end
    end

    %Cut matrix ends and starts
    matrix=matrix(index_start:index_end,:);



    %Interpolate matrix to homogenize the time between entries
    if subject_step == 1
        time = matrix(:,1); % is the starting vector of time
        %Interpolate to have continuous time intervals for filtering
        new_time = time(1):Time_step:time(end); % new vector of time with
fixed dt=1/fs (Time_step)
        matrixTemp(:,1)=new_time;
        for i=2:size(matrix,2)
            matrixTemp(:,i) =
interp1(matrix(:,1),matrix(:,i),new_time,'linear','extrap');
        end
    else
        for i=2:size(matrix,2)
            matrixTemp(:,i) =
interp1(matrix(:,1),matrix(:,i),new_time,'linear','extrap');
        end
    end
    matrix=matrixTemp;

    %%%%%%%%%%%%%%%%%%%%%%%%%%%%%%%%%%%%%%%%%%%%%%%%%%%%%%%%%%%%%%%%%%%%%%
    % Approximating angles from accelerometer sensor data, as opposed to
    % the upper extremity, unstable sensor readings are not
    % - A very small constant (10^-17) is added to the denominator of the
formula, so
    % that it can never reach zero and produce NaNs.
```

```
    constant = 0.00000000000000001;
    % - Taking the absolute value of the entry in the denominator enables
not
    % having to use a complicated case distinction for the atan formula.
    %Value of target joint calculated with arcsin() and case distinction

    %online in the source code of Reha-Stim Medtech AG (formerly known as
    %YouRehab), which we have no access to.
    value_TAR = matrix(:,2);
    target = matrix(:,3);

    % Trunk, always contralateral of target joint
    % Lateral trunk atan(x/y) because atan-peak not in starting position
    % Ventral trunk atan(z/y) because atan-peak not in starting position
    switch SIDE
      case 0 % if joint on the left side is played
        trunk_lat =
rad2deg(atan(matrix(:,16)./(sqrt(matrix(:,17).^2)+constant)));
        trunk_vent =
rad2deg(atan(matrix(:,18)./(sqrt(matrix(:,17).^2)+constant)));
      case 1 % if joint on the right side is played
        trunk_lat =
rad2deg(atan(matrix(:,28)./(sqrt(matrix(:,29).^2)+constant)));
        trunk_vent =
rad2deg(atan(matrix(:,30)./(sqrt(matrix(:,29).^2)+constant)));
    end

    switch HIP % flexion (y/z), rotation (x/z)
      case 0 %Hip NOT target joint
             % Hip sensors chosen for both because KneeM moves with knee
        hipR =
rad2deg(atan(matrix(:,14)./(sqrt(matrix(:,15).^2)+constant)));
        hipL =
rad2deg(atan(matrix(:,26)./(sqrt(matrix(:,27).^2)+constant)));
        hipRotR =
rad2deg(atan(matrix(:,13)./(sqrt(matrix(:,15).^2)+constant)));
        hipRotL =
rad2deg(atan(matrix(:,25)./(sqrt(matrix(:,27).^2)+constant)));
      case 1 %Hip is target joint
             % Knee sensors chosen because move less with hip movements
        hipR =
rad2deg(atan(matrix(:,23)./(sqrt(matrix(:,24).^2)+constant))); %value
inserted later anyway
        hipL =
rad2deg(atan(matrix(:,35)./(sqrt(matrix(:,36).^2)+constant))); %value
inserted later anyway
        hipRotR =
rad2deg(atan(matrix(:,22)./(sqrt(matrix(:,24).^2)+constant)));
        hipRotL =
rad2deg(atan(matrix(:,34)./(sqrt(matrix(:,36).^2)+constant)));
    end

    %Other angle approximations that aren't dependent on hip as target
    %joint
        kneeR =
rad2deg(atan(matrix(:,23)./(sqrt(matrix(:,24).^2)+constant)))-
rad2deg(atan(matrix(:,20)./(sqrt(matrix(:,21).^2)+constant)));
        kneeL =
rad2deg(atan(matrix(:,35)./(sqrt(matrix(:,36).^2)+constant)))-
rad2deg(atan(matrix(:,32)./(sqrt(matrix(:,33).^2)+constant)));
```

```
        footR =
rad2deg(atan(matrix(:,41)./(sqrt(matrix(:,42).^2)+constant)))-
rad2deg(atan(matrix(:,38)./(sqrt(matrix(:,39).^2)+constant)));
        footL =
rad2deg(atan(matrix(:,47)./(sqrt(matrix(:,48).^2)+constant)))-
rad2deg(atan(matrix(:,44)./(sqrt(matrix(:,45).^2)+constant)));

%%%%%%%%%%%%%%%%%%%%%%%%%%%%%%%%%%%%%%%%%%%%%%%%%%%%%%%%%%%%%%%%%%%%%%%%%%%

    disp(filename);

    %Filter data

        value_TAR_filt(:,subject_step) = filtfilt(b,a,value_TAR);
        target_filt(:,subject_step) = filtfilt(b,a,target);

        trunk_lat_filt(:,subject_step) = filtfilt(b,a,trunk_lat);
        trunk_vent_filt(:,subject_step) = filtfilt(b,a,trunk_vent);
        hipR_filt(:,subject_step) = filtfilt(b,a,hipR);
        hipL_filt(:,subject_step) = filtfilt(b,a,hipL);
        hipRotR_filt(:,subject_step) = filtfilt(b,a,hipRotR);
        hipRotL_filt(:,subject_step) = filtfilt(b,a,hipRotL);
        kneeR_filt(:,subject_step) = filtfilt(b,a,kneeR);
        kneeL_filt(:,subject_step) = filtfilt(b,a,kneeL);
        footR_filt(:,subject_step) = filtfilt(b,a,footR);
        footL_filt(:,subject_step) = filtfilt(b,a,footL);


    %Calculating derivative (change value/delta) of filtered signals
        delta_trunk_lat(:,subject_step) =
[diff(trunk_lat_filt(:,subject_step))]/Time_step;
        delta_trunk_vent(:,subject_step) =
[diff(trunk_vent_filt(:,subject_step))]/Time_step;
        delta_hipR(:,subject_step) =
[diff(hipR_filt(:,subject_step))]/Time_step;
        delta_hipL(:,subject_step) =
[diff(hipL_filt(:,subject_step))]/Time_step;
        delta_hipRotR(:,subject_step) =
[diff(hipRotR_filt(:,subject_step))]/Time_step;
        delta_hipRotL(:,subject_step) =
[diff(hipRotL_filt(:,subject_step))]/Time_step;
        delta_kneeR(:,subject_step) =
[diff(kneeR_filt(:,subject_step))]/Time_step;
        delta_kneeL(:,subject_step) =
[diff(kneeL_filt(:,subject_step))]/Time_step;
        delta_footR(:,subject_step) =
[diff(footR_filt(:,subject_step))]/Time_step;
        delta_footL(:,subject_step) =
[diff(footL_filt(:,subject_step))]/Time_step;
end


%% Values deleted from REFERENCE matrix because not coincide with movement
%Also, all HCH and P are adjusted/deleted here!
switch JOINT
    case 1
    %--Hip R
delta_footR(:,[10,53,64,88,71,95,72,97,74,98,99,102,84,85]) = NaN; %HA10,
HCH22, P01, P01R, P08, P08R, P09, P10R, P11, P11R, P12R, P15R, P21, P22
delta_trunk_lat(1150:1350,93) = NaN; %P06R using hand to lift leg
delta_trunk_vent(1150:1350,93) = NaN; %P06R using hand to lift leg
```

```
delta_hipR(1150:1350,93) = NaN; %P06R using hand to lift leg
delta_hipL(1150:1350,93) = NaN; %P06R using hand to lift leg
delta_hipRotR(1150:1350,93) = NaN; %P06R using hand to lift leg
delta_hipRotL(1150:1350,93) = NaN; %P06R using hand to lift leg
delta_kneeR(1150:1350,93) = NaN; %P06R using hand to lift leg
delta_kneeL(1150:1350,93) = NaN; %P06R using hand to lift leg
delta_footR(1150:1350,93) = NaN; %P06R using hand to lift leg
delta_footL(1150:1350,93) = NaN; %P06R using hand to lift leg
value_TAR_filt(660:end,71) = NaN; %P06R using hand to lift leg
delta_trunk_lat(1:70,103) = NaN; %P16R touches Hip Master sensor
delta_trunk_vent(1:70,103) = NaN; %P16R touches Hip Master sensor
delta_kneeR(940:1949,103) = NaN; %P16R touches Knee Master sensor

    case 2
    %--Hip L
delta_footR(:,[10,53,64,88,71,95,72,97,74,98,99,100,102,84,85]) = NaN;
%HA10, HCH22, P01, P01R, P08, P08R, P09, P10R, P11, P11R, P12R, P13R, P15R,
P21, P22
delta_trunk_lat(300:540,52) = NaN; % HCH21 touches sensor
delta_trunk_vent(300:540,52) = NaN; % HCH21 touches sensor
delta_trunk_lat(660:end,71) = NaN; %P08 using hand to lift leg
delta_trunk_vent(660:end,71) = NaN; %P08 using hand to lift leg
delta_hipR(660:end,71) = NaN; %P08 using hand to lift leg
delta_hipL(660:end,71) = NaN; %P08 using hand to lift leg
delta_hipRotR(660:end,71) = NaN; %P08 using hand to lift leg
delta_hipRotL(660:end,71) = NaN; %P08 using hand to lift leg
delta_kneeR(660:end,71) = NaN; %P08 using hand to lift leg
delta_kneeL(660:end,71) = NaN; %P08 using hand to lift leg
delta_footR(660:end,71) = NaN; %P08 using hand to lift leg
delta_footL(660:end,71) = NaN; %P08 using hand to lift leg
value_TAR_filt(660:end,71) = NaN; %P08 using hand to lift leg

    case 3
%--Knee R
delta_footR(:,[10,53,64,88,89,71,95,72,97,74,98,99,100,102,84,85,86]) =
NaN; %HA10, HCH22, P01, P01R, P02R, P08, P08R, P09, P10R, P11, P11R, P12R,
P13R, P15R, P21, P22, P23
delta_trunk_lat(680:800,16) = NaN; % HA16 laughing
delta_hipRotR(680:800,51) = NaN; %HCH20 waving
delta_hipR(680:800,51) = NaN; %HCH20 waving

    case 4
%--Knee L
delta_footR(:,[10,53,64,88,89,71,95,72,97,74,98,99,100,102,84,85,86]) =
NaN; %HA10, HCH22, P01, P01R, P02R, P08, P08R, P09, P10R, P11, P11R, P12R,
P13R, P15R, P21, P22, P23
delta_hipRotR(1:200,76) = NaN; %P13 touches sensor
delta_hipRotR(480:600,42) = NaN; %HCH11 touches sensor
delta_hipR(480:600,42) = NaN; %HCH11 touches sensor
    case 5
%--Foot R
delta_hipRotL(540:640,10) = NaN; %HA10 touches sensor
delta_hipL(540:640,10) = NaN; %HA10 touches sensor
delta_trunk_lat(570:870,67) = NaN; % P4 touches sensor
    case 6
%--Foot L
delta_footR(:,[10,53,97,74,98,99,100,102,83,84]) = NaN; %HA10, HCH22, P10R,
P11, P11R, P12R, P13R, P15R, P20, P21
delta_hipRotR(1300:1500,49) = NaN; %HCH18 touches sensor
delta_hipR(1300:1500,49) = NaN; %HCH18 touches sensor
end
```

```
%% Calculating the mean and SD

for subject_step=1:length(Choose_index)

        %%%%%%% For all the joints %%%%%%%%%
        %Init temp matrix
        delta_trunk_lat_temp = delta_trunk_lat;
        delta_trunk_vent_temp = delta_trunk_vent;
        delta_hipR_temp = delta_hipR;
        delta_hipL_temp = delta_hipL;
        delta_hipRotR_temp = delta_hipRotR;
        delta_hipRotL_temp = delta_hipRotL;
        delta_kneeR_temp = delta_kneeR;
        delta_kneeL_temp = delta_kneeL;
        delta_footR_temp = delta_footR;
        delta_footL_temp = delta_footL;



        %Delete current subject as well as P and HCH which aren't reference
group
        % (exclude form reference SD and mean) in temp matrix
        delta_trunk_lat_temp =
reference_matrix(delta_trunk_lat_temp,subject_step,REF_HA);
        delta_trunk_vent_temp =
reference_matrix(delta_trunk_vent_temp,subject_step,REF_HA);
        delta_hipR_temp =
reference_matrix(delta_hipR_temp,subject_step,REF_HA);
        delta_hipL_temp =
reference_matrix(delta_hipL_temp,subject_step,REF_HA);
        delta_hipRotR_temp =
reference_matrix(delta_hipRotR_temp,subject_step,REF_HA);
        delta_hipRotL_temp =
reference_matrix(delta_hipRotL_temp,subject_step,REF_HA);
        delta_kneeR_temp =
reference_matrix(delta_kneeR_temp,subject_step,REF_HA);
        delta_kneeL_temp =
reference_matrix(delta_kneeL_temp,subject_step,REF_HA);
        delta_footR_temp =
reference_matrix(delta_footR_temp,subject_step,REF_HA);
        delta_footL_temp =
reference_matrix(delta_footL_temp,subject_step,REF_HA);

        %Calculate SD
        SD_delta_trunk_lat(:,subject_step) =
nanstd(delta_trunk_lat_temp,0,2);
        SD_delta_trunk_vent(:,subject_step) =
nanstd(delta_trunk_vent_temp,0,2);
        SD_delta_hipR(:,subject_step) = nanstd(delta_hipR_temp,0,2);
        SD_delta_hipL(:,subject_step) = nanstd(delta_hipL_temp,0,2);
        SD_delta_hipRotR(:,subject_step) = nanstd(delta_hipRotR_temp,0,2);
        SD_delta_hipRotL(:,subject_step) = nanstd(delta_hipRotL_temp,0,2);
        SD_delta_kneeR(:,subject_step) = nanstd(delta_kneeR_temp,0,2);
        SD_delta_kneeL(:,subject_step) = nanstd(delta_kneeL_temp,0,2);
        SD_delta_footR(:,subject_step) = nanstd(delta_footR_temp,0,2);
        SD_delta_footL(:,subject_step) = nanstd(delta_footL_temp,0,2);

        %Calculate absolute errors -> (subject value- mean[w/o
subject])/SD[w/o subject]
        error_delta_trunk_lat(:,subject_step) =
(delta_trunk_lat(:,subject_step)-
nanmean(delta_trunk_lat_temp,2))./SD_delta_trunk_lat(:,subject_step);
```

```
        error_delta_trunk_vent(:,subject_step) =
(delta_trunk_vent(:,subject_step)-
nanmean(delta_trunk_vent_temp,2))./SD_delta_trunk_vent(:,subject_step);
        error_delta_hipR(:,subject_step) = (delta_hipR(:,subject_step)-
nanmean(delta_hipR_temp,2))./SD_delta_hipR(:,subject_step);
        error_delta_hipL(:,subject_step) = (delta_hipL(:,subject_step)-
nanmean(delta_hipL_temp,2))./SD_delta_hipL(:,subject_step);
        error_delta_hipRotR(:,subject_step) =
(delta_hipRotR(:,subject_step)-
nanmean(delta_hipRotR_temp,2))./SD_delta_hipRotR(:,subject_step);
        error_delta_hipRotL(:,subject_step) =
(delta_hipRotL(:,subject_step)-
nanmean(delta_hipRotL_temp,2))./SD_delta_hipRotL(:,subject_step);
        error_delta_kneeR(:,subject_step) = (delta_kneeR(:,subject_step)-
nanmean(delta_kneeR_temp,2))./SD_delta_kneeR(:,subject_step);
        error_delta_kneeL(:,subject_step) = (delta_kneeL(:,subject_step)-
nanmean(delta_kneeL_temp,2))./SD_delta_kneeL(:,subject_step);
        error_delta_footR(:,subject_step) = (delta_footR(:,subject_step)-
nanmean(delta_footR_temp,2))./SD_delta_footR(:,subject_step);
        error_delta_footL(:,subject_step) = (delta_footL(:,subject_step)-
nanmean(delta_footL_temp,2))./SD_delta_footL(:,subject_step);

        %Mean of all absolute errors per subject
        A_trunk_lat = nanmean(sqrt(error_delta_trunk_lat.^2));
        A_trunk_vent = nanmean(sqrt(error_delta_trunk_vent.^2));
        A_hipR = nanmean(sqrt(error_delta_hipR.^2));
        A_hipL = nanmean(sqrt(error_delta_hipL.^2));
        A_hipRotR = nanmean(sqrt(error_delta_hipRotR.^2));
        A_hipRotL = nanmean(sqrt(error_delta_hipRotL.^2));
        A_kneeR = nanmean(sqrt(error_delta_kneeR.^2));
        A_kneeL = nanmean(sqrt(error_delta_kneeL.^2));
        A_footR = nanmean(sqrt(error_delta_footR.^2));
        A_footL = nanmean(sqrt(error_delta_footL.^2));

        %%%%%%% For the target joint %%%%%%%%%

        %Init temp matrix
        value_TAR_filt_temp = value_TAR_filt;
        %Creating reference matrix with only HA in it
        value_TAR_filt_temp =
reference_matrix(value_TAR_filt_temp,subject_step,REF_HA);
        %Calculate SD with specified mean
        SD_value_TAR_filt(:,subject_step) =
std_spec_mean(value_TAR_filt_temp,target_filt(:,subject_step));
        %Calculate errors wrt STD
        value_TAR_filt_error(:,subject_step) =
(value_TAR_filt(:,subject_step)-
target_filt(:,subject_step))./SD_value_TAR_filt(:,subject_step);
        %Mean of errors for target joint
        A_TAR_joint = nanmean(sqrt(value_TAR_filt_error.^2));



        %Matrix containing TARGET joint and co-movements in columns
        no_col_names =
[A_TAR_joint',A_trunk_lat',A_trunk_vent',A_hipR',A_hipL',A_hipRotR',A_hipRo
tL',A_kneeR',A_kneeL',A_footR',A_footL'];
        colNames =
{'TAR_joint','trunk_lat','trunk_vent','hipR','hipL','hipRotR','hipRotL','kn
eeR','kneeL','footR','footL'};
        A_A = array2table(no_col_names,'VariableNames',colNames);
end
```

Algorithm of the upper extremity: Matlab code

```
% This algorithm uses data from accelerometers to approximate joint angles
% which occur as involuntary movements as part a target tracking task.

clear all; close all;
debug_out=0;

% Don't forget to change JOINT depending on whether TARjoint is on left or
right side
JOINT = 10; %ShR=1, ShL=2, ElbR=3, ElbL=4, psR=5, psL=6, wrR=7, wrL=8,
fingR=9, fingL=10
if  mod(JOINT,2)== 2 %Required for switching of trunk movement sensor
    SIDE = 1; %odd number indicating that target joint is on right side
else
    SIDE = 0; %even number indicating that target joint is on left side
end
REF_HA = 33; %number of healthy adults as reference group
%In certain positions the approximated angles are not stable, therefore a
%cutoff value is defined after which point the angles are discarded.
cutoff = 20; %cutoff value for critital sensor positions in [degrees]
reference_limit = 20; %reference values needed to calculate an error
(distance from mean_ref/SD_ref)

%time inbetween 2 measurments after interpolating
Time_step = 0.015; %Chosen so that no information is lost because shortest
interval in output files 0.017s
fc = 1.5; fs = 1/Time_step; %frequency of measurments

%Definition of the lowpass filter
[b,a] = butter(6,fc/(fs/2),'low');

%Files to be analyzed
Choose_index =
{'1','2','3','4','5','6','7','8','9','10','11','12','13','14','15','16','17
','18','19','20','21','22','23','24','25','26','27','28','29','30','31','32
','33','C1','C2','C3','C4','C5','C6','C7','C8','C9','C10','C11','C12','C13'
,'C14','C15','C16','C17','C18','C19','C20','C21','C22','C23','C24','C25','C
26','C27','C28','C29','C30','C31','C32','P1','P2','P3','P4','P5','P6','P7',
'P8','P9','P10','P11','P12','P13','P14','P15','P16','P17','P18','P19','P20'
,'P21','P22','P23','P24','P25','P26','P27','P28','P29','P30','P31','zp01','
zp02','zp03','zp04','zp05','zp06','zp07','zp08','zp09','zp10','zp11','zp12'
,'zp13','zp14','zp15','P1R','P2R','P3R','P4R','P5R','P6R','P7R','P8R','P9R'
,'P10R','P11R','P12R','P13R','P14R','P15R','P16R','P17R','P18R','P19R','P20
R','P21R','P22R','P23R','P24R','P25R','P26R','P27R','P28R','P29R','P30R','P
31R','zp01R','zp02R','zp03R','zp04R','zp05R','zp06R','zp07R','zp08R','zp09R
','zp10R','zp11R','zp12R','zp13R','zp14R','zp15R'};



for subject_step=1:length(Choose_index)

    info1=dir('*.csv');
    for i=1:size(info1,1)
        filename=info1(i).name;
        if
strcmp(filename(1:length(Choose_index{subject_step})),Choose_index{subject_
step});
            break;
        end
        if i==size(info1,1)
            error('No file found')
        end
```

```
    end

    fid = fopen(filename,'r');
    C = textscan(fid, repmat('%s',1,48), 'delimiter',';',
'CollectOutput',true);
    fclose(fid);

    %Read .csv file to predefined length of the file
    matrix= [];
    matrix= dlmread(filename,';',[1 0 size(C{1},1)-2 47]);

    %Assigning target and value paths
    target = matrix(:,3); %target path defined by stars
    value = matrix(:,2); %path flown by player

    %Cutting target vector to find relevant phase - Start of target path
    stopcondition=0;
    for i=2:length(target)
        if target(i)~=target(i-1) && stopcondition==0
            index_start=i;
            stopcondition=1;
        end
    end

    %Cutting target vector to find relevant phase - End of target path
    stopcondition=0;
    for i=length(target):-1:2
        if target(i)~=target(i-1) && stopcondition==0
            index_end=i;
            stopcondition=1;
        end
    end

    %Cut matrix from start to end index
    matrix=matrix(index_start:index_end,:);

    %Interpolate matrix to homogenize the time between entries (for
    %filtering)
    if subject_step == 1
        time = matrix(:,1); % is the starting vector of time
        new_time = time(1):Time_step:time(end); % new vector of time with
fixed dt=1/fs (Time_step)
        matrixTemp(:,1)=new_time;
        for i=2:size(matrix,2)
            matrixTemp(:,i) =
interp1(matrix(:,1),matrix(:,i),new_time,'linear','extrap');
        end
    else
        for i=2:size(matrix,2)
            matrixTemp(:,i) =
interp1(matrix(:,1),matrix(:,i),new_time,'linear','extrap');
        end
    end
    matrix=matrixTemp;
    %%%%%%%%%%%%%%%%%%%%%%%%%%%%%%%%%%%%%%%%%%%%%%%%%%%%%%%%%%%%%%%%%%%%%%
    % Approximating angles from accelerometer sensor data
    % - A very small constant (10^-17) is added to the denominator of the
formula, so
    % that it can never reach zero and produce NaNs.
    constant = 0.00000000000000001;
```

```
    % - Taking the absolute value of the entry in the denominator enables
not
    % having to use a complicated case distinction for the atan formula.

    %Value of target joint calculated with arcsin() and case distinction
    %online in the source code of Reha-Stim Medtech AG (formerly known as
    %YouRehab), which we have no access to.
    target_cut(:,subject_step) = matrix(:,3);
    value_cut(:,subject_step) = matrix(:,2);

    %Trunk angles depending on side played, NaNs not expected because
    %participant sits as upright as possible.
    % Ventral trunk atan(x/z) because atan-peak not in starting position
    % Lateral trunk atan(y/z) because atan-peak not in starting position
    switch SIDE
        case 0 %if LEFT joint played the right sensor is reference
            trunk_lat(:,subject_step) =
rad2deg(atan(matrix(:,23)./(sqrt(matrix(:,24).^2)+constant)));
            trunk_vent(:,subject_step) =
rad2deg(atan(matrix(:,22)./(sqrt(matrix(:,24).^2)+constant)));

        case 1 %if RIGHT joint played the left sensor is reference
            trunk_lat(:,subject_step) =
rad2deg(atan(matrix(:,35)./(sqrt(matrix(:,36).^2)+constant)));
            trunk_vent(:,subject_step) =
rad2deg(atan(matrix(:,34)./(sqrt(matrix(:,36).^2)+constant)));
    end

    % For the other joints general formula is "Master angle (M) - Slave
angle (S)"
    % Shoulder Abduction angle - no unstable angles because player upright
    Sh_abdR(:,subject_step) =
rad2deg(atan(matrix(:,23)./(sqrt(matrix(:,24).^2)+constant)))-
rad2deg(atan(matrix(:,20)./(sqrt(matrix(:,21).^2)+constant)));
    Sh_abdL(:,subject_step) =
rad2deg(atan(matrix(:,35)./(sqrt(matrix(:,36).^2)+constant)))-
rad2deg(atan(matrix(:,32)./(sqrt(matrix(:,33).^2)+constant)));

    %Shoulder Rotation if arm not hanging at side
    % Master sensor vertical not anticipated
    Sh_rotR(:,subject_step) =
rad2deg(atan(matrix(:,24)./(sqrt(matrix(:,22).^2)+constant)))-
rad2deg(atan(matrix(:,21)./(sqrt(matrix(:,19).^2)+constant)));
    Sh_rotL(:,subject_step) =
rad2deg(atan(matrix(:,36)./(sqrt(matrix(:,34).^2)+constant)))-
rad2deg(atan(matrix(:,33)./(sqrt(matrix(:,31).^2)+constant)));

    % if arms hangig at side value will be NaN
    for datastep = 1:size(matrix,1)
        if
abs(rad2deg(atan(matrix(datastep,20)./(sqrt(matrix(datastep,21).^2+matrix(d
atastep,19).^2))))) >= (90-cutoff)
            Sh_rotR(datastep) =      NaN;
        end

        if
abs(rad2deg(atan(matrix(datastep,32)./(sqrt(matrix(datastep,33).^2+matrix(d
atastep,31).^2))))) >= (90-cutoff)
            Sh_rotL(datastep) =      NaN;
        end
    end
```

```
    %Elbow flexion/extension (further explanations at site of formula)
    % 1) if My=0 and MyROT (z=0) is smaller than cutoff deg -> NaN
    % 2) if Sy=0 and SyROT (z=0) is smaller than cutoff deg -> NaN
    % 3) if both sensors are horizontal irrespective of ROT-> NaN
    % Problem: z and y are both almost zero. This only happens, when
    % shoulder is abducted.
    % The fact that the elbow slave sensor rotates
    % only minimally with pro/supination of lower arm and prevents z from
    % being close to zero when y =0 but shoulder not abducted
    % (normal flexion of elbow with arm hangig at side).

    Elb_R(:,subject_step)=
rad2deg(atan(matrix(:,41)./(sqrt(matrix(:,42).^2)+constant)))-
rad2deg(atan(matrix(:,38)./(sqrt(matrix(:,39).^2)+constant)));
    Elb_L(:,subject_step)=
rad2deg(atan(matrix(:,47)./(sqrt(matrix(:,48).^2)+constant)))-
rad2deg(atan(matrix(:,44)./(sqrt(matrix(:,45).^2)+constant)));

    for datastep= 1:size(matrix,1)
        %RIGHT elbow
        % 1) if My horizontal and MyROT close to z=0
        if
(abs(rad2deg(atan(matrix(datastep,41)./(sqrt(matrix(datastep,42).^2+matrix(
datastep,40).^2))))) < cutoff) &&
(abs(rad2deg(atan(matrix(datastep,42)./(sqrt(matrix(datastep,40).^2+constan
t))))) < cutoff)
            Elb_R(datastep,subject_step) =         NaN;

        end
        % 2) if Sy horizontal and SyROT close to z=0
        if
(abs(rad2deg(atan(matrix(datastep,38)./(sqrt(matrix(datastep,39).^2+matrix(
datastep,37)^2))))) < cutoff) &&
(abs(rad2deg(atan(matrix(datastep,39)./(sqrt(matrix(datastep,37).^2+constan
t))))) < cutoff)
           Elb_R(datastep,subject_step) =         NaN;

        end
        % 3) if Sy and My horzontal then NaN
        if
(abs(rad2deg(atan(matrix(datastep,38)./(sqrt(matrix(datastep,39).^2+matrix(
datastep,37).^2))))) < cutoff) &&
(abs(rad2deg(atan(matrix(datastep,41)./(sqrt(matrix(datastep,42).^2+matrix(
datastep,40).^2))))) < cutoff)
            Elb_R(datastep,subject_step) =         NaN;
        end

        %LEFT elbow
        % 1) if My horizontal and MyROT close to z=0
        if
abs(rad2deg(atan(matrix(datastep,47)./(sqrt(matrix(datastep,48).^2+matrix(d
atastep,46).^2)))))  < cutoff &&
abs(rad2deg(atan(matrix(datastep,48)./(sqrt(matrix(datastep,46).^2+constant
))))) < cutoff
%             Elb_L(datastep,subject_step) =
rad2deg(atan(matrix(datastep,47)./(sqrt(matrix(datastep,48).^2+matrix(datas
tep,46).^2))))-
rad2deg(atan(matrix(datastep,44)./(sqrt(matrix(datastep,45).^2)+constant)))
;
            Elb_L(datastep,subject_step) =         NaN;
```

```
        end
        % 2) if Sy horizontal and SyROT close to z=0
        if
abs(rad2deg(atan(matrix(datastep,44)./(sqrt(matrix(datastep,45).^2+matrix(d
atastep,43).^2))))) < cutoff &&
abs(rad2deg(atan(matrix(datastep,45)./(sqrt(matrix(datastep,43).^2+constant
))))) < cutoff
%             Elb_L(datastep,subject_step) =
rad2deg(atan(matrix(datastep,47)./(sqrt(matrix(datastep,48).^2)+constant)))
-
rad2deg(atan(matrix(datastep,44)./(sqrt(matrix(datastep,45).^2+matrix(datas
tep,43).^2))));
            Elb_L(datastep,subject_step) =         NaN;
        end
        % 3) if Sy and My horzontal then NaN
        if
abs(rad2deg(atan(matrix(datastep,44)./(sqrt(matrix(datastep,45).^2+matrix(d
atastep,43).^2))))) < cutoff &&
abs(rad2deg(atan(matrix(datastep,47)./(sqrt(matrix(datastep,48).^2+matrix(d
atastep,46).^2))))) < cutoff
            Elb_L(datastep,subject_step) =         NaN;
        end
    end

    %LA pro-/supination is NaN
    % if one of the two sensors y-axis is larger than 90-cutoff
    LA_psR(:,subject_step) =
rad2deg(atan(matrix(:,39)./(sqrt(matrix(:,37).^2)+constant)))+rad2deg(atan(
matrix(:,10)./(sqrt(matrix(:,8).^2)+constant)));
    LA_psL(:,subject_step) =
rad2deg(atan(matrix(:,45)./(sqrt(matrix(:,43).^2)+constant)))+rad2deg(atan(
matrix(:,7)./(sqrt(matrix(:,5).^2)+constant)));
    for datastep=1:size(matrix,1)
        if
abs(rad2deg(atan(matrix(datastep,38)./(sqrt(matrix(datastep,39).^2+matrix(d
atastep,37).^2))))) >= (90-cutoff) ||
abs((rad2deg(atan(matrix(datastep,9)./(sqrt(matrix(datastep,10).^2+matrix(d
atastep,8).^2)))))) >= (90-cutoff);
            LA_psR(datastep,subject_step) =        NaN;
        end
        if
abs(rad2deg(atan(matrix(datastep,44)./(sqrt(matrix(datastep,45).^2+matrix(d
atastep,43).^2))))) >= (90-cutoff) ||
abs((rad2deg(atan(matrix(datastep,6)./(sqrt(matrix(datastep,7).^2+matrix(da
tastep,5).^2)))))) >= (90-cutoff);
            LA_psL(datastep,subject_step) =        NaN;
        end
    end

    %Wrist extentsion flexion is NaN
    % 1) if My is horizontal and MyROT is smaller than cutoff deg -> M:
3axis sol
    % 2) if Sy is horizontal and SyROT is smaller than cutoff deg -> NaN
      % Because if Master sensor is non-horizontal we're looking at ADD/ABD
      % of wrist
    % 3) if both sensors horizontal (y=0) and BOTH z components =0 -> NaN
      % both sensors y-axes horizontally aligned is acceptable since
      % adduction/abduction of wrist is not measured REDUNDANT
    % BUT as soon as one sensors y-axis is horizontal and the z-axis comes
    % close to zero the calculated angle is too strongly influenced by
    % y-axis rotation (z component being close to zero)
```

```
    Wr_flexR(:,subject_step) =
rad2deg(atan(matrix(:,38)./(sqrt(matrix(:,39).^2)+constant)))-
rad2deg(atan(matrix(:,9)./(sqrt(matrix(:,10).^2)+constant)));
    Wr_flexL(:,subject_step) =
rad2deg(atan(matrix(:,44)./(sqrt(matrix(:,45).^2)+constant)))-
rad2deg(atan(matrix(:,6)./(sqrt(matrix(:,7).^2)+constant)));

    for datastep=1:size(matrix,1)
        %RIGHT wrist
        %if My horizontal and MyROT close to z=0 then 3axis solution for M
        %because 6/10 conditions require player to lay arms on table
        if
abs(rad2deg(atan(matrix(datastep,38)./(sqrt(matrix(datastep,39).^2+matrix(d
atastep,37).^2))))) < cutoff &&
abs(rad2deg(atan(matrix(datastep,39)./(sqrt(matrix(datastep,37).^2+constant
))))) < cutoff
            Wr_flexR(datastep,subject_step) =
rad2deg(atan(matrix(datastep,38)./(sqrt(matrix(datastep,39).^2+matrix(datas
tep,37).^2))))-
rad2deg(atan(matrix(datastep,9)./(sqrt(matrix(datastep,10).^2)+constant)));
        end
        %if Sy horizontal and SyROT close to z=0
        if
abs(rad2deg(atan(matrix(datastep,9)./(sqrt(matrix(datastep,10).^2+matrix(da
tastep,8).^2))))) < cutoff &&
abs(rad2deg(atan(matrix(datastep,10)./(sqrt(matrix(datastep,8).^2+constant)
)))) < cutoff
            Wr_flexR(datastep,subject_step) =      NaN;
        end

        %LEFT wrist
        %if My horizontal and MyROT close to z=0 then 3axis solution for M
        %because 6/10 conditions require player to lay arms on table
        if
abs(rad2deg(atan(matrix(datastep,44)./(sqrt(matrix(datastep,45.)^2+matrix(d
atastep,43).^2))))) < cutoff &&
abs(rad2deg(atan(matrix(datastep,45)./(sqrt(matrix(datastep,43).^2+constant
))))) < cutoff
            Wr_flexL(datastep,subject_step) =
rad2deg(atan(matrix(datastep,44)./(sqrt(matrix(datastep,45).^2+matrix(datas
tep,43).^2))))-
rad2deg(atan(matrix(datastep,6)./(sqrt(matrix(datastep,7).^2)+constant)));
        end
        %if Sy horizontal and MyROT close to z=0
        if
abs(rad2deg(atan(matrix(datastep,6)./(sqrt(matrix(datastep,7).^2+matrix(dat
astep,5).^2))))) < cutoff &&
abs(rad2deg(atan(matrix(datastep,7)./(sqrt(matrix(datastep,5).^2+constant))
))) < cutoff;
            Wr_flexL(datastep,subject_step) =      NaN;
        end
    end

    %Finger flexion extension with bend sensors of YouGrabber system
    Finger_R(:,subject_step) =      matrix(:,12);
    Finger_L(:,subject_step) =      matrix(:,11);


%%%%%%%%%%%%%%%%%%%%%%%%%%%%%%%%%%%%%%%%%%%%%%%%%%%%%%%%%%%%%%%%%%%%%%%%%%%

    disp(filename);
```

```
    %Remove unstable sections (NaNs) and store in index to reuse later, not
for
    % TARvalue and Finger
    % trunk_lat NaNs not expected, index1
    % trunk_vent NaNs not expected, index2
    % Shoulder abduction

    Sh_rotR_temp=Sh_rotR(:,subject_step);
    index5=find(isnan(Sh_rotR_temp)==1);
    Sh_rotR_temp(index5)=[];

    Sh_rotL_temp=Sh_rotL(:,subject_step);
    index6=find(isnan(Sh_rotL_temp)==1);
    Sh_rotL_temp(index6)=[];

    Elb_R_temp=Elb_R(:,subject_step);
    index7=find(isnan(Elb_R_temp)==1);
    Elb_R_temp(index7)=[];

    Elb_L_temp=Elb_L(:,subject_step);
    index8=find(isnan(Elb_L_temp)==1);
    Elb_L_temp(index8)=[];

    LA_psR_temp=LA_psR(:,subject_step);
    index9=find(isnan(LA_psR_temp)==1);
    LA_psR_temp(index9)=[];

    LA_psL_temp=LA_psL(:,subject_step);
    index10=find(isnan(LA_psL_temp)==1);
    LA_psL_temp(index10)=[];

    Wr_flexR_temp=Wr_flexR(:,subject_step);
    index11=find(isnan(Wr_flexR_temp)==1);
    Wr_flexR_temp(index11)=[];

    Wr_flexL_temp=Wr_flexL(:,subject_step);
    index12=find(isnan(Wr_flexL_temp)==1);
    Wr_flexL_temp(index12)=[];

    %Filtering the data
    % temporary matrix is filtered and 'padded' with zeros to match the
    % matrix length (resulting in zeros at the end of the array)
    % - Value, target and Finger_R/L are always the same length, also trunk
    % shoulder abduction are not expectet to contain NaNs and thus are same
    % length
    % - Wrist and Elbow are extremely unlikely to ever be empty (only NaNs
    % present) because of the position needed to create NaNs
    if isempty(Sh_rotR_temp) == 1
        Sh_rotR_filt(1:size(Sh_rotR,1),subject_step) = NaN;
        Sh_rotR_temp = NaN;
        index5(end)=[];
    elseif size(Sh_rotR_temp,1) <19
        Sh_rotR_filt(:,subject_step) = [Sh_rotR_temp;zeros(size(matrix,1)-
size(Sh_rotR_temp,1),1)];
    else
        Sh_rotR_filt(:,subject_step) =
[filtfilt(b,a,Sh_rotR_temp);zeros(size(matrix,1)-size(Sh_rotR_temp,1),1)];
    end
    if isempty(Sh_rotL_temp) == 1
        Sh_rotL_filt(1:size(Sh_rotL,1),subject_step) = NaN;
```

```
        Sh_rotL_temp = NaN;
        index6(end)=[];
    elseif size(Sh_rotL_temp,1) <19
        Sh_rotL_filt(:,subject_step) = [Sh_rotL_temp;zeros(size(matrix,1)-
size(Sh_rotL_temp,1),1)];
    else
        Sh_rotL_filt(:,subject_step) =
[filtfilt(b,a,Sh_rotL_temp);zeros(size(matrix,1)-size(Sh_rotL_temp,1),1)];
    end

    if isempty(Elb_R_temp) == 1
        Elb_R_filt(1:size(Elb_R,1),subject_step) = NaN;
        Elb_R_temp = NaN;
        index7(end)=[];
    elseif size(Elb_R_temp,1) <19
        Elb_R_filt(:,subject_step) = [Elb_R_temp;zeros(size(matrix,1)-
size(Elb_R_temp,1),1)];
    else
        Elb_R_filt(:,subject_step) =
[filtfilt(b,a,Elb_R_temp);zeros(size(matrix,1)-size(Elb_R_temp,1),1)];
    end
    if isempty(Elb_L_temp) == 1
        Elb_L_filt(1:size(Elb_L,1),subject_step) = NaN;
        Elb_L_temp = NaN;
        index8(end)=[];
    elseif size(Elb_L_temp,1) <19
        Elb_L_filt(:,subject_step) = [Elb_L_temp;zeros(size(matrix,1)-
size(Elb_L_temp,1),1)];
    else
        Elb_L_filt(:,subject_step) =
[filtfilt(b,a,Elb_L_temp);zeros(size(matrix,1)-size(Elb_L_temp,1),1)];
    end

    if isempty(LA_psR_temp)
        LA_psR_filt(1:size(LA_psR,1),subject_step) = NaN;
        LA_psR_temp = NaN;
        index9(end)=[];
    elseif size(LA_psR_temp,1) <19
        LA_psR_filt(:,subject_step) = [LA_psR_temp;zeros(size(matrix,1)-
size(LA_psR_temp,1),1)];
    else
        LA_psR_filt(:,subject_step) =
[filtfilt(b,a,LA_psR_temp);zeros(size(matrix,1)-size(LA_psR_temp,1),1)];
    end
    if isempty(LA_psL_temp)
        LA_psL_filt(1:size(LA_psL,1),subject_step) = NaN;
        LA_psL_temp = NaN;
        index10(end)=[];
    elseif size(LA_psL_temp,1) <19
        LA_psL_filt(:,subject_step) = [LA_psL_temp;zeros(size(matrix,1)-
size(LA_psL_temp,1),1)];
    else
        LA_psL_filt(:,subject_step) =
[filtfilt(b,a,LA_psL_temp);zeros(size(matrix,1)-size(LA_psL_temp,1),1)];
    end

    if isempty(Wr_flexR_temp)
        Wr_flexR_filt(1:size(Wr_flexR,1),subject_step) = NaN;
        Wr_flexR_temp = NaN;
        index11(end)=[];
    elseif size(Wr_flexR_temp,1) <19
```

```
        Wr_flexR_filt(:,subject_step) =
[Wr_flexR_temp;zeros(size(matrix,1)-size(Wr_flexR_temp,1),1)];
    else
        Wr_flexR_filt(:,subject_step) =
[filtfilt(b,a,Wr_flexR_temp);zeros(size(matrix,1)-
size(Wr_flexR_temp,1),1)];
    end
    if isempty(Wr_flexL_temp)
        Wr_flexL_filt(1:size(Wr_flexL,1),subject_step) = NaN;
        Wr_flexL_temp = NaN;
        index12(end)=[];
    elseif size(Wr_flexL_temp,1) <19
        Wr_flexL_filt(:,subject_step) =
[Wr_flexL_temp;zeros(size(matrix,1)-size(Wr_flexL_temp,1),1)];
    else
        Wr_flexL_filt(:,subject_step) =
[filtfilt(b,a,Wr_flexL_temp);zeros(size(matrix,1)-
size(Wr_flexL_temp,1),1)];
    end

        value_TAR_filt(:,subject_step) =
filtfilt(b,a,value_cut(:,subject_step));
        target_filt(:,subject_step) =
filtfilt(b,a,target_cut(:,subject_step));

        trunk_lat_filt(:,subject_step) =
filtfilt(b,a,trunk_lat(:,subject_step));
        trunk_vent_filt(:,subject_step) =
filtfilt(b,a,trunk_vent(:,subject_step));
        Sh_abdR_filt(:,subject_step) =
filtfilt(b,a,Sh_abdR(:,subject_step));
        Sh_abdL_filt(:,subject_step) =
filtfilt(b,a,Sh_abdL(:,subject_step));
        Finger_R_filt(:,subject_step) =
filtfilt(b,a,Finger_R(:,subject_step));
        Finger_L_filt(:,subject_step) =
filtfilt(b,a,Finger_L(:,subject_step));


    %Calculating derivative (delta value/delta time) of filtered signals
    % but only for the data that was  filtered, zero padding ignored
    % a NaN is added after the derivative to have same length for delta as
    % for angle value (index can now never be longer than delta because NaN
    % added)
    % in the end zero padding is added again to store the data
    delta_trunk_lat(:,subject_step) =
[diff(trunk_lat_filt(:,subject_step)); NaN]/Time_step;
    delta_trunk_vent(:,subject_step)
=[diff(trunk_vent_filt(:,subject_step));NaN]/Time_step;
    delta_Sh_abdR(:,subject_step) = [diff(Sh_abdR_filt(:,subject_step));
NaN]/Time_step;
    delta_Sh_abdL(:,subject_step) = [diff(Sh_abdL_filt(:,subject_step));
NaN]/Time_step;
    delta_Sh_rotR(:,subject_step) =
[[diff(Sh_rotR_filt(1:length(Sh_rotR_temp),subject_step)); NaN]/Time_step;
zeros(length(matrix)-length(Sh_rotR_temp),1)];
    delta_Sh_rotL(:,subject_step) =
[[diff(Sh_rotL_filt(1:length(Sh_rotL_temp),subject_step)); NaN]/Time_step;
zeros(length(matrix)-length(Sh_rotL_temp),1)];
    delta_Elb_R(:,subject_step) =
[[diff(Elb_R_filt(1:length(Elb_R_temp),subject_step)); NaN]/Time_step;
zeros(length(matrix)-length(Elb_R_temp),1)];
```

```
    delta_Elb_L(:,subject_step) =
[[diff(Elb_L_filt(1:length(Elb_L_temp),subject_step)); NaN]/Time_step;
zeros(length(matrix)-length(Elb_L_temp),1)];
    delta_LA_psR(:,subject_step) =
[[diff(LA_psR_filt(1:length(LA_psR_temp),subject_step)); NaN]/Time_step;
zeros(length(matrix)-length(LA_psR_temp),1)];
    delta_LA_psL(:,subject_step) =
[[diff(LA_psL_filt(1:length(LA_psL_temp),subject_step)); NaN]/Time_step;
zeros(length(matrix)-length(LA_psL_temp),1)];
    delta_Wr_flexR(:,subject_step) =
[[diff(Wr_flexR_filt(1:length(Wr_flexR_temp),subject_step));
NaN]/Time_step; zeros(length(matrix)-length(Wr_flexR_temp),1)];
    delta_Wr_flexL(:,subject_step) =
[[diff(Wr_flexL_filt(1:length(Wr_flexL_temp),subject_step));
NaN]/Time_step; zeros(length(matrix)-length(Wr_flexL_temp),1)];
    delta_Finger_R(:,subject_step) = [diff(Finger_R_filt(:,subject_step));
NaN]/Time_step;
    delta_Finger_L(:,subject_step) = [diff(Finger_L_filt(:,subject_step));
NaN]/Time_step;

    %This section uses a function that adds the NaNs back in the location
    %they were before the extraction, thus all values have same positions
as at
    %beginning
    delta_Sh_rotR_NaNs =
insertAt(delta_Sh_rotR(1:length(Sh_rotR_temp),subject_step),index5);
    delta_Sh_rotR(:,subject_step) = delta_Sh_rotR_NaNs;

    delta_Sh_rotL_NaNs =
insertAt(delta_Sh_rotL(1:length(Sh_rotL_temp),subject_step),index6);
    delta_Sh_rotL(:,subject_step) = delta_Sh_rotL_NaNs;

    delta_Elb_R_NaNs =
insertAt(delta_Elb_R(1:length(Elb_R_temp),subject_step),index7);
    delta_Elb_R(:,subject_step) = delta_Elb_R_NaNs;

    delta_Elb_L_NaNs =
insertAt(delta_Elb_L(1:length(Elb_L_temp),subject_step),index8);
    delta_Elb_L(:,subject_step) = delta_Elb_L_NaNs;

    delta_LA_psR_NaNs =
insertAt(delta_LA_psR(1:length(LA_psR_temp),subject_step),index9);
    delta_LA_psR(:,subject_step) = delta_LA_psR_NaNs;

    delta_LA_psL_NaNs =
insertAt(delta_LA_psL(1:length(LA_psL_temp),subject_step),index10);
    delta_LA_psL(:,subject_step) = delta_LA_psL_NaNs;

    delta_Wr_flexR_NaNs =
insertAt(delta_Wr_flexR(1:length(Wr_flexR_temp),subject_step),index11);
    delta_Wr_flexR(:,subject_step) = delta_Wr_flexR_NaNs;

    delta_Wr_flexL_NaNs =
insertAt(delta_Wr_flexL(1:length(Wr_flexL_temp),subject_step),index12);
    delta_Wr_flexL(:,subject_step) = delta_Wr_flexL_NaNs;
end

%---Values deleted from REFERENCE matrix because not coincide with movement
%Also, all healthy children and patients sensor recordings that failed are
adjusted/deleted here!
```

```
switch JOINT
    case 1
%--Shoulder R
delta_Sh_rotL(:,15) = NaN(length(delta_Sh_rotL(:,15)),1); %HA15
delta_Sh_rotL(:,25) = NaN(length(delta_Sh_rotL(:,25)),1); %HA25

    case 2
%--Shoulder L
delta_Elb_R(:,25) = NaN(length(delta_Elb_R(:,25)),1); %HA25, elbR
delta_LA_psR(:,25) = NaN(length(delta_LA_psR(:,25)),1); %HA25, psR
delta_Wr_flexR(:,25) = NaN(length(delta_Wr_flexR(:,25)),1); %HA25, WrR
delta_Elb_R(:,28) = NaN(length(delta_Elb_R(:,28)),1); %HA28, elbR
delta_LA_psR(:,28) = NaN(length(delta_LA_psR(:,28)),1); %HA28, psR
delta_Wr_flexR(:,28) = NaN(length(delta_Wr_flexR(:,28)),1); %HA28, WrR

    case 3
%--Elbow R
delta_Wr_flexR(:,16) = NaN(length(delta_Wr_flexR(:,16)),1); %HA16
delta_LA_psL(:,20) = NaN(length(delta_LA_psL(:,20)),1); %HA20

    case 4
%--Elbow L
delta_Sh_rotR(:,1) = NaN(length(delta_Sh_rotR(:,1)),1); %HA1
delta_Wr_flexR(:,34) = NaN(length(delta_Wr_flexR(:,34)),1); %HCH1
delta_Wr_flexR(:,69) = NaN(length(delta_Wr_flexR(:,69)),1); %P9

    case 5
%--LA pro-/supination R
delta_Elb_L(:,44) = NaN(length(delta_Elb_L(:,44)),1); %HCH11
delta_LA_psL(1:1200,110) = NaN(length(delta_Elb_L(1:1200,110)),1); %P14R
delta_Elb_L(:,96) = NaN(length(delta_Elb_L(:,96)),1); %P31
delta_Elb_R(:,96) = NaN(length(delta_Elb_R(:,96)),1); %P31

    case 6
%--LA pro-/supination L
delta_Wr_flexR(1:180,41) = NaN(length(delta_Wr_flexR(1:180,41)),1); %HCH8
delta_Elb_R(1:180,41) = NaN(length(delta_Elb_R(1:180,41)),1); %HCH8
delta_Elb_L(:,120) = NaN(length(delta_Elb_L(:,120)),1); %P24R
delta_Elb_L(:,96) = NaN(length(delta_Elb_L(:,96)),1); %P31
delta_Elb_R(:,96) = NaN(length(delta_Elb_R(:,96)),1); %P31

    case 7
%--Wrist flex R
delta_Elb_L(:,44) = NaN(length(delta_Elb_L(:,44)),1); %HCH11
delta_Elb_R(:,61) = NaN(length(delta_Elb_R(:,61)),1); %HCH28
delta_Elb_R(:,96) = NaN(length(delta_Elb_R(:,96)),1); %P31

    case 8
%--Wrist flex L
delta_Elb_R(:,74) = NaN(length(delta_Elb_R(:,74)),1); %HCH28
delta_Elb_R(:,96) = NaN(length(delta_Elb_R(:,96)),1); %P31
delta_Elb_R(:,28) = NaN(length(delta_Elb_R(:,28)),1); %HA25, elbR
delta_LA_psR(:,28) = NaN(length(delta_LA_psR(:,28)),1); %HA25, psR
delta_Wr_flexR(:,28) = NaN(length(delta_Wr_flexR(:,28)),1); %HA25, wrR

    case 9
%-- Finger R
delta_Elb_L(:,96) = NaN(length(delta_Elb_L(:,96)),1); %P31

    case 10
%--Finger L
```

```
delta_trunk_vent(600:1000,12) =
NaN(length(delta_trunk_vent(600:1000,12)),1); %HA12
delta_trunk_lat(600:1000,12) =
NaN(length(delta_trunk_vent(600:1000,12)),1); %HA12
delta_Sh_abdR(600:1000,12) = NaN(length(delta_trunk_vent(600:1000,12)),1);
%HA12
delta_Sh_abdL(600:1000,12) = NaN(length(delta_trunk_vent(600:1000,12)),1);
%HA12
delta_Sh_rotR(600:1000,12) = NaN(length(delta_trunk_vent(600:1000,12)),1);
%HA12
delta_Sh_rotL(600:1000,12) = NaN(length(delta_trunk_vent(600:1000,12)),1);
%HA12
delta_Elb_R(600:1000,12) = NaN(length(delta_trunk_vent(600:1000,12)),1);
%HA12
delta_Elb_L(600:1000,12) = NaN(length(delta_trunk_vent(600:1000,12)),1);
%HA12
delta_LA_psR(600:1000,12) = NaN(length(delta_trunk_vent(600:1000,12)),1);
%HA12
delta_LA_psL(600:1000,12) = NaN(length(delta_trunk_vent(600:1000,12)),1);
%HA12
delta_Finger_R(600:1000,12) = NaN(length(delta_trunk_vent(600:1000,12)),1);
%HA12
delta_Elb_R(:,96) = NaN(length(delta_Elb_R(:,96)),1); %P31
delta_Wr_flexR(:,96) = NaN(length(delta_Wr_flexR(:,96)),1); %P31
end

%% Calculating the mean and standard deviations (relate all participants
%  to healthy adults)

for subject_step=1:length(Choose_index)

    %%%%%%% For all the joints %%%%%%%%%
    %Initiate temporary matrix
    delta_trunk_lat_temp = delta_trunk_lat;
    delta_trunk_vent_temp = delta_trunk_vent;
    delta_Sh_abdR_temp = delta_Sh_abdR;
    delta_Sh_abdL_temp = delta_Sh_abdL;
    delta_Sh_rotR_temp = delta_Sh_rotR;
    delta_Sh_rotL_temp = delta_Sh_rotL;
    delta_Elb_R_temp = delta_Elb_R;
    delta_Elb_L_temp = delta_Elb_L;
    delta_LA_psR_temp = delta_LA_psR;
    delta_LA_psL_temp = delta_LA_psL;
    delta_Wr_flexR_temp = delta_Wr_flexR;
    delta_Wr_flexL_temp = delta_Wr_flexL;
    delta_Finger_R_temp = delta_Finger_R;
    delta_Finger_L_temp = delta_Finger_L;

    %Delete current subject (if healthy adult) as well as patients and
    %  healthy children which aren't reference group
    % (exclude form reference SD and mean) in temp matrix
    delta_trunk_lat_temp =
reference_matrix(delta_trunk_lat_temp,subject_step,REF_HA);
    delta_trunk_vent_temp =
reference_matrix(delta_trunk_vent_temp,subject_step,REF_HA);
    delta_Sh_abdR_temp =
reference_matrix(delta_Sh_abdR_temp,subject_step,REF_HA);
    delta_Sh_abdL_temp =
reference_matrix(delta_Sh_abdL_temp,subject_step,REF_HA);
    delta_Sh_rotR_temp =
reference_matrix(delta_Sh_rotR_temp,subject_step,REF_HA);
```

```
    delta_Sh_rotL_temp =
reference_matrix(delta_Sh_rotL_temp,subject_step,REF_HA);
    delta_Elb_R_temp =
reference_matrix(delta_Elb_R_temp,subject_step,REF_HA);
    delta_Elb_L_temp =
reference_matrix(delta_Elb_L_temp,subject_step,REF_HA);
    delta_LA_psR_temp =
reference_matrix(delta_LA_psR_temp,subject_step,REF_HA);
    delta_LA_psL_temp =
reference_matrix(delta_LA_psL_temp,subject_step,REF_HA);
    delta_Wr_flexR_temp =
reference_matrix(delta_Wr_flexR_temp,subject_step,REF_HA);
    delta_Wr_flexL_temp =
reference_matrix(delta_Wr_flexL_temp,subject_step,REF_HA);
    delta_Finger_R_temp =
reference_matrix(delta_Finger_R_temp,subject_step,REF_HA);
    delta_Finger_L_temp =
reference_matrix(delta_Finger_L_temp,subject_step,REF_HA);

    %Calculate SD ignoring NaNs, only if 'reference_limit' or more
reference values
    % the case of missing reference values has to be expected in Sh_rot,
    % Elb, LA_ps, and Wr_flex
    SD_delta_trunk_lat(:,subject_step) = nanstd(delta_trunk_lat_temp,0,2);
    SD_delta_trunk_vent(:,subject_step) =
nanstd(delta_trunk_vent_temp,0,2);
    SD_delta_Sh_abdR(:,subject_step) = nanstd(delta_Sh_abdR_temp,0,2);
    SD_delta_Sh_abdL(:,subject_step) = nanstd(delta_Sh_abdL_temp,0,2);

    for data_step=1:size(delta_Sh_rotR_temp,1)
        if sum(~isnan(delta_Sh_rotR_temp(data_step,:))) >= reference_limit
        SD_delta_Sh_rotR(data_step,subject_step) =
nanstd(delta_Sh_rotR_temp(data_step,:),0,2);
        else
        SD_delta_Sh_rotR(data_step,subject_step) = NaN;
        end
    end
    for data_step=1:size(delta_Sh_rotL_temp,1)
        if sum(~isnan(delta_Sh_rotL_temp(data_step,:))) >= reference_limit
        SD_delta_Sh_rotL(data_step,subject_step) =
nanstd(delta_Sh_rotL_temp(data_step,:),0,2);
        else
        SD_delta_Sh_rotL(data_step,subject_step) = NaN;
        end
    end
    for data_step=1:size(delta_Elb_R_temp,1)
        if sum(~isnan(delta_Elb_R_temp(data_step,:))) >= reference_limit
        SD_delta_Elb_R(data_step,subject_step) =
nanstd(delta_Elb_R_temp(data_step,:),0,2);
        else
        SD_delta_Elb_R(data_step,subject_step) = NaN;
        end
    end
    for data_step=1:size(delta_Elb_L_temp,1)
        if sum(~isnan(delta_Elb_L_temp(data_step,:))) >= reference_limit
        SD_delta_Elb_L(data_step,subject_step) =
nanstd(delta_Elb_L_temp(data_step,:),0,2);
        else
        SD_delta_Elb_L(data_step,subject_step) = NaN;
        end
    end
    for data_step=1:size(delta_LA_psR_temp,1)
```

```
        if sum(~isnan(delta_LA_psR_temp(data_step,:))) >= reference_limit
        SD_delta_LA_psR(data_step,subject_step) = 
nanstd(delta_LA_psR_temp(data_step,:),0,2);
        else
        SD_delta_LA_psR(data_step,subject_step) = NaN;
        end
    end
    for data_step=1:size(delta_LA_psL_temp,1)
        if sum(~isnan(delta_LA_psL_temp(data_step,:))) >= reference_limit
        SD_delta_LA_psL(data_step,subject_step) = 
nanstd(delta_LA_psL_temp(data_step,:),0,2);
        else
        SD_delta_LA_psL(data_step,subject_step) = NaN;
        end
    end
    for data_step=1:size(delta_Wr_flexR_temp,1)
        if sum(~isnan(delta_Wr_flexR_temp(data_step,:))) >= reference_limit
        SD_delta_Wr_flexR(data_step,subject_step) = 
nanstd(delta_Wr_flexR_temp(data_step,:),0,2);
        else
        SD_delta_Wr_flexR(data_step,subject_step) = NaN;
        end
    end
    for data_step=1:size(delta_Wr_flexL_temp,1)
        if sum(~isnan(delta_Wr_flexL_temp(data_step,:))) >= reference_limit
        SD_delta_Wr_flexL(data_step,subject_step) = 
nanstd(delta_Wr_flexL_temp(data_step,:),0,2);
        else
        SD_delta_Wr_flexL(data_step,subject_step) = NaN;
        end
    end

    SD_delta_Finger_R(:,subject_step) = nanstd(delta_Finger_R_temp,0,2);
    SD_delta_Finger_L(:,subject_step) = nanstd(delta_Finger_L_temp,0,2);

    %Calculating weights for usage in final analysis (only shoulder will
    % have combined outcome, the combination of abduction and rotation)
    % WEIGHTS: all entries that are not NaNs+1/total entries
    %   Total of ~NaNs from subject and reference group taken (Numerator)
    %   +1 has to be added because before a NaN was added (calculating
    %   derivative, e.g. delta_Sh_rotR)
    %   (delta and SD_delta difference, bacause NaNs can come from both
    %    vectors and subtracting both vectors results in a new one with
    %    zeros and NaNS -> NaNs if either one of both vectors elements is
NaN)
    WEIGHT_Sh_rotR(subject_step,:) = 
(sum(~isnan(delta_Sh_rotR(:,subject_step)-
SD_delta_Sh_rotR(:,subject_step)))+1)/size(delta_Sh_rotR,1);
    WEIGHT_Sh_rotL(subject_step,:) = 
(sum(~isnan(delta_Sh_rotL(:,subject_step)-
SD_delta_Sh_rotL(:,subject_step)))+1)/size(delta_Sh_rotL,1);

    %Calculate absolute errors ignoring NaNs, (subject value- mean[w/o 
subject])/SD[w/o subject]
    error_delta_trunk_lat(:,subject_step) = 
(delta_trunk_lat(:,subject_step)-
nanmean(delta_trunk_lat_temp,2))./SD_delta_trunk_lat(:,subject_step);
    error_delta_trunk_vent(:,subject_step) = 
(delta_trunk_vent(:,subject_step)-
nanmean(delta_trunk_vent_temp,2))./SD_delta_trunk_vent(:,subject_step);
    error_delta_Sh_abdR(:,subject_step) = (delta_Sh_abdR(:,subject_step)-
nanmean(delta_Sh_abdR_temp,2))./SD_delta_Sh_abdR(:,subject_step);
```

```
    error_delta_Sh_abdL(:,subject_step) = (delta_Sh_abdL(:,subject_step)-
nanmean(delta_Sh_abdL_temp,2))./SD_delta_Sh_abdL(:,subject_step);
    error_delta_Sh_rotR(:,subject_step) = (delta_Sh_rotR(:,subject_step)-
nanmean(delta_Sh_rotR_temp,2))./SD_delta_Sh_rotR(:,subject_step);
    error_delta_Sh_rotL(:,subject_step) = (delta_Sh_rotL(:,subject_step)-
nanmean(delta_Sh_rotL_temp,2))./SD_delta_Sh_rotL(:,subject_step);
    error_delta_Elb_R(:,subject_step) = (delta_Elb_R(:,subject_step)-
nanmean(delta_Elb_R_temp,2))./SD_delta_Elb_R(:,subject_step);
    error_delta_Elb_L(:,subject_step) = (delta_Elb_L(:,subject_step)-
nanmean(delta_Elb_L_temp,2))./SD_delta_Elb_L(:,subject_step);
    error_delta_LA_psR(:,subject_step) = (delta_LA_psR(:,subject_step)-
nanmean(delta_LA_psR_temp,2))./SD_delta_LA_psR(:,subject_step);
    error_delta_LA_psL(:,subject_step) = (delta_LA_psL(:,subject_step)-
nanmean(delta_LA_psL_temp,2))./SD_delta_LA_psL(:,subject_step);
    error_delta_Wr_flexR(:,subject_step) = (delta_Wr_flexR(:,subject_step)-
nanmean(delta_Wr_flexR_temp,2))./SD_delta_Wr_flexR(:,subject_step);
    error_delta_Wr_flexL(:,subject_step) = (delta_Wr_flexL(:,subject_step)-
nanmean(delta_Wr_flexL_temp,2))./SD_delta_Wr_flexL(:,subject_step);
    error_delta_Finger_R(:,subject_step) = (delta_Finger_R(:,subject_step)-
nanmean(delta_Finger_R_temp,2))./SD_delta_Finger_R(:,subject_step);
    error_delta_Finger_L(:,subject_step) = (delta_Finger_L(:,subject_step)-
nanmean(delta_Finger_L_temp,2))./SD_delta_Finger_L(:,subject_step);

    %Mean of all absolute errors per subject
    A_trunk_lat = nanmean(sqrt(error_delta_trunk_lat.^2),1);
    A_trunk_vent = nanmean(sqrt(error_delta_trunk_vent.^2),1);
    A_Sh_abdR = nanmean(sqrt(error_delta_Sh_abdR.^2),1);
    A_Sh_abdL = nanmean(sqrt(error_delta_Sh_abdL.^2),1);
    A_Sh_rotR = nanmean(sqrt(error_delta_Sh_rotR.^2),1);
    A_Sh_rotL = nanmean(sqrt(error_delta_Sh_rotL.^2),1);
    A_Elb_R = nanmean(sqrt(error_delta_Elb_R.^2),1);
    A_Elb_L = nanmean(sqrt(error_delta_Elb_L.^2),1);
    A_LA_psR = nanmean(sqrt(error_delta_LA_psR.^2),1);
    A_LA_psL = nanmean(sqrt(error_delta_LA_psL.^2),1);
    A_Wr_flexR = nanmean(sqrt(error_delta_Wr_flexR.^2),1);
    A_Wr_flexL = nanmean(sqrt(error_delta_Wr_flexL.^2),1);
    A_Finger_R = nanmean(sqrt(error_delta_Finger_R.^2),1);
    A_Finger_L = nanmean(sqrt(error_delta_Finger_L.^2),1);

    %%%%%%% For the target joint %%%%%%%%%

    %Initiating temporary matrix
    value_TAR_filt_temp = value_TAR_filt;
    %Creating reference vector from reference matrix
    value_TAR_filt_temp =
reference_matrix(value_TAR_filt_temp,subject_step,REF_HA);
    %Calculate SD with specified "mean" (target trajectory)
    SD_value_TAR_filt(:,subject_step) =
std_spec_mean(value_TAR_filt_temp,target_filt(:,subject_step));
    %Calculate errors wrt STD
    value_TAR_filt_error(:,subject_step) = (value_TAR_filt(:,subject_step)-
target_filt(:,subject_step))./SD_value_TAR_filt(:,subject_step);
    %Mean of errors for target joint
    A_TAR_joint = mean(sqrt(value_TAR_filt_error.^2));



    %Matrix containing TARGET joint and co-movements in columns
    no_col_names =
[A_TAR_joint',A_trunk_lat',A_trunk_vent',A_Sh_abdR',A_Sh_abdL',A_Sh_rotR',W
EIGHT_Sh_rotR,A_Sh_rotL',WEIGHT_Sh_rotL,A_Elb_R',A_Elb_L',A_LA_psR',A_LA_ps
L',A_Wr_flexR',A_Wr_flexL',A_Finger_R',A_Finger_L'];
```

```
    colNames =
{'TAR_joint','trunk_lat','trunk_vent','Sh_abdR','Sh_abdL','Sh_rotR','WEIGHT
_Sh_rotR','Sh_rotL','WEIGHT_Sh_rotL','Elb_R','Elb_L','LA_psR','LA_psL','Wr_
flexR','Wr_flexL','Finger_R','Finger_L'};
    A_A = array2table(no_col_names,'VariableNames',colNames);
End
```

Support function 1

```
function origVec = insertAt(vector,index)
%If variable index has an NO entry (no NaNs) the given vector is the same
%as the original vector (no insertion of NaNs)
%ELSE:
%This function iserts NaNs at the specified positions (index) of the vector
%given.
    c=false(1,length(vector) + length(index));

    if length(index) < 1
        origVec = vector;
    else

    c(index) = true;
    result = nan(size(c));
    result(~c) = vector;
    origVec = result;
    end
end
```

Support function 2

```
function ref_matrix =
reference_matrix(temp_matrix,subject_step,reference_HA)
 % This function generates the reference matirx for the analysis of the
 % subject_step
    if subject_step <= reference_HA %subject analysed is an adult
        temp_matrix(:,reference_HA+1:end) = []; %deletes all colums with
HCH and P &
        temp_matrix(:,subject_step) = []; %deletes the current subject
    else %subject is a healthy child or patient
        temp_matrix(:,reference_HA+1:end) = []; %deletes all HCH and P
    end
    ref_matrix = temp_matrix;
end
```

Support function 3

```
function SD=std_spec_mean(data,spec_mean)
%Function to get a standard deviation with specified mean
%(not necessarily mean of data provided)
for step=1:size(data,1)
    SD(step) = sqrt(1/(sum(~isnan(data(step,:)))-1)*nansum((data(step,:)-
spec_mean(step)).^2));
end
```
